# Association of ambient air pollutant mixtures with IVF/ICSI-ET clinical pregnancy rates during critical exposure periods

**DOI:** 10.1093/hropen/hoae051

**Published:** 2024-09-06

**Authors:** Rui-Ling Liu, Tong Wang, Ying-Ling Yao, Xing-Yu Lv, Yu-Ling Hu, Xin-Zhen Chen, Xiao-Jun Tang, Zhao-Hui Zhong, Li-Juan Fu, Xin Luo, Li-Hong Geng, Shao-Min Yu, Yu-Bin Ding

**Affiliations:** Department of Obstetrics and Gynecology, Women and Children’s Hospital of Chongqing Medical University, Chongqing, China; Department of Toxicology, Joint International Research Laboratory of Reproduction and Development of the Ministry of Education of China, School of Public Health, Chongqing Medical University, Chongqing, China; Department of Toxicology, Key Lab of Medical Protection for Electromagnetic Radiation, Ministry of Education of China, Institute of Toxicology, College of Preventive Medicine, Third Military Medical University (Army Medical University), Chongqing, China; Department of Toxicology, Joint International Research Laboratory of Reproduction and Development of the Ministry of Education of China, School of Public Health, Chongqing Medical University, Chongqing, China; The Reproductive Center, Sichuan Jinxin Xinan Women & Children’s Hospital, Chengdu, Sichuan, China; The Reproductive Center, Sichuan Jinxin Xinan Women & Children’s Hospital, Chengdu, Sichuan, China; Department of Obstetrics and Gynecology, Women and Children’s Hospital of Chongqing Medical University, Chongqing, China; Department of Toxicology, Joint International Research Laboratory of Reproduction and Development of the Ministry of Education of China, School of Public Health, Chongqing Medical University, Chongqing, China; Department of Toxicology, Joint International Research Laboratory of Reproduction and Development of the Ministry of Education of China, School of Public Health, Chongqing Medical University, Chongqing, China; Department of Toxicology, Joint International Research Laboratory of Reproduction and Development of the Ministry of Education of China, School of Public Health, Chongqing Medical University, Chongqing, China; Department of Pharmacology, Academician Workstation, Changsha Medical University, Changsha, China; Department of Obstetrics and Gynecology, The First Affiliated Hospital of Chongqing Medical University, Chongqing, China; The Reproductive Center, Sichuan Jinxin Xinan Women & Children’s Hospital, Chengdu, Sichuan, China; Department of Obstetrics and Gynecology, The People’s Hospital of Yubei, Chongqing, China; Department of Obstetrics and Gynecology, Women and Children’s Hospital of Chongqing Medical University, Chongqing, China; Department of Pharmacology, Academician Workstation, Changsha Medical University, Changsha, China

**Keywords:** ART, mixture pollutants, specific exposure periods, clinical pregnancy, quantile g-computation, Bayesian kernel machine regression

## Abstract

**STUDY QUESTION:**

Does exposure to a mixture of ambient air pollutants during specific exposure periods influence clinical pregnancy rates in women undergoing IVF/ICSI-embryo transfer (ET) cycles?

**SUMMARY ANSWER:**

The specific exposure period from ET to the serum hCG test was identified as a critical exposure window as exposure to sulfur dioxide (SO_2_) or a combination of air pollutants was associated with a decreased likelihood of clinical pregnancy.

**WHAT IS KNOWN ALREADY:**

Exposure to a single pollutant may impact pregnancy outcomes in women undergoing ART. However, in daily life, individuals often encounter mixed pollution, and limited research exists on the effects of mixed air pollutants and the specific exposure periods.

**STUDY DESIGN, SIZE, DURATION:**

This retrospective cohort study involved infertile patients who underwent their initial IVF/ICSI-ET cycle at an assisted reproduction center between January 2020 and January 2023. Exclusions were applied for patients meeting specific criteria, such as no fresh ET, incomplete clinical and address information, residency outside the 17 cities in the Sichuan Basin, age over 45 years, use of donor semen, thin endometrium (<8 mm) and infertility factors unrelated to tubal or ovulation issues. In total, 5208 individuals were included in the study.

**PARTICIPANTS/MATERIALS, SETTING, METHODS:**

Daily average levels of six air pollutants (fine particulate matter (PM_2.5_), inhalable particulate matter (PM_10_), SO_2_, nitrogen dioxide (NO_2_), carbon monoxide (CO), and ozone (O_3_)) were acquired from air quality monitoring stations. The cumulative average levels of various pollutants were determined using the inverse distance weighting (IDW) method across four distinct exposure periods (Period 1: 90 days before oocyte retrieval; Period 2: oocyte retrieval to ET; Period 3: ET to serum hCG test; Period 4: 90 days before oocyte retrieval to serum hCG test). Single-pollutant logistic regression, two-pollutant logistic regression, Quantile g-computation (QG-C) regression, and Bayesian kernel machine regression (BKMR) were employed to evaluate the influence of pollutants on clinical pregnancy rates. Stratified analyses were executed to discern potentially vulnerable populations.

**MAIN RESULTS AND THE ROLE OF CHANCE:**

The clinical pregnancy rate for participants during the study period was 54.53%. Single-pollutant logistic models indicated that for PM_2.5_ during specific exposure Period 1 (adjusted odds ratio [aOR] = 0.83, 95% CI: 0.70–0.99) and specific exposure Period 4 (aOR = 0.83, 95% CI: 0.69–0.98), and SO_2_ in specific exposure Period 3 (aOR = 0.92, 95% CI: 0.86–0.99), each interquartile range (IQR) increment exhibited an association with a decreased probability of clinical pregnancy. Consistent results were observed with dual air pollution models. In the multi-pollution analysis, QG-C indicated a 12% reduction in clinical pregnancy rates per IQR increment of mixed pollutants during specific exposure Period 3 (aOR = 0.89, 95% CI: 0.79–0.99). Among these pollutants, SO_2_ (33.40%) and NO_2_ (33.40%) contributed the most to the negative effects. The results from BKMR and QG-C were consistent. Stratified analysis revealed increased susceptibility to ambient air pollution among individuals who underwent transfer of two embryos, those with BMI ≥ 24 kg/m^2^ and those under 35 years old.

**LIMITATIONS, REASONS FOR CAUTION:**

Caution was advised in interpreting the results due to the retrospective nature of the study, which was prone to selection bias from non-random sampling. Smoking and alcohol, known confounding factors in IVF/ICSI-ET, were not accounted for. Only successful cycles that reached the hCG test were included, excluding a few patients who did not reach the ET stage. While IDW was used to estimate pollutant concentrations at residential addresses, data on participants’ work locations and activity patterns were not collected, potentially affecting the accuracy of exposure prediction.

**WIDER IMPLICATIONS OF THE FINDINGS:**

Exposure to a mixture of pollutants, spanning from ET to the serum hCG test (Period 3), appeared to be correlated with a diminished probability of achieving clinical pregnancy. This association suggested a potential impact of mixed pollutants on the interaction between embryos and the endometrium, as well as embryo implantation during this critical stage, potentially contributing to clinical pregnancy failure. This underscored the importance of providing women undergoing ART with comprehensive information to comprehend the potential environmental influences and motivating them to adopt suitable protective measures when feasible, thereby mitigating potential adverse effects of contaminants on reproductive health.

**STUDY FUNDING/COMPETING INTEREST(S):**

This work received support from the National Key Research and Development Program of China (No. 2023YFC2705900), the National Natural Science Foundation of China (Nos. 82171664, 81971391, 82171668), the Natural Science Foundation of Chongqing Municipality of China (Nos. CSTB2022NSCQ-LZX0062, CSTB2023TIAD-KPX0052) and the Foundation of State Key Laboratory of Ultrasound in Medicine and Engineering (No. 2021KFKT013). The authors report no conflicts of interest.

**TRIAL REGISTRATION NUMBER:**

N/A.

WHAT DOES THIS MEAN FOR PATIENTS? With assisted reproductive technology (ART), exposure to air pollutants can affect the chances of a clinical pregnancy. However, how air pollutants affect pregnancy outcomes during different periods of the ART cycle is unclear, especially given that patients may be exposed to multiple pollutants at the same time. This study was conducted to investigate how exposure to a combination of air pollutants during specific time periods affects clinical pregnancy rates in patients undergoing ART treatment. Our findings indicated that pollutant exposure at the time period between embryo transfer and the serum human chorionic gonadotropin (hCG) test was a critical phase for reducing the likelihood of clinical pregnancy. Exposure to sulfur dioxide (SO_2_) or mixed air pollutants during this specific exposure period was associated with a decreased likelihood of clinical pregnancy. Furthermore, individuals who underwent the transfer of two embryos, those with body mass index (BMI) ≥24 kg/m^2^, and those under 35 years of age were found to be more vulnerable to the adverse effects of ambient air pollution. This underscores the importance of providing women undergoing ART with comprehensive information to enhance their understanding of potential environmental impacts. Encouraging them to adopt suitable protective measures, where feasible, is crucial in minimizing potential adverse effects of environmental contamination on reproductive health.

## Introduction

Assisted reproduction technologies, particularly IVF/ICSI-embryo transfer (ET), have transformed the landscape of reproductive medicine, providing couples with a viable solution to navigate challenges related to infertility (Zegers-Hochschild *et al.*, 2017). Despite the advancements in IVF/ICSI-ET, its success remains influenced by various enigmatic factors. In recent years, there has been increasing attention on understanding the potential impact of ambient air pollutants on IVF/ICSI-ET outcomes (Choe *et al.*, 2018; Boulet *et al.*, 2019). Epidemiological investigations have identified ambient air pollution, encompassing fine inhalable particulate matter (PM_2.5_), coarse particulate matter (PM_10_), carbon monoxide (CO), nitrogen dioxide (NO_2_), ozone (O_3_), and sulfur dioxide (SO_2_), as potential risk factors for infertility and adverse pregnancy outcomes. Conditions such as miscarriage, stillbirth, preterm birth, gestational diabetes, and preeclampsia have been associated with exposure to these pollutants (Padula *et al.*, 2019; Gaskins *et al.*, 2020; Hu *et al.*, 2020a; Gaskins *et al.*, 2021; deSouza *et al.*, 2022; Liang *et al.*, 2023).

In the realm of IVF/ICSI-ET, where the precise timing of exposure is vital due to meticulously scheduled events in the IVF/ICSI-ET cycle, the relationship between ambient air pollution exposure during IVF/ICSI-ET cycles and conception rates remains uncertain (Choe *et al.*, 2018; Boulet *et al.*, 2019; Shi *et al.*, 2021; Wu *et al.*, 2021; Liu *et al.*, 2022). This uncertainty may be attributed to variations in region, population characteristics, methodologies for exposure estimation, and specific exposure periods.

Research across Chinese cities and provinces have linked ambient air pollution to IVF/ICSI-ET pregnancy outcomes (Qiu *et al.*, 2019; Li *et al.*, 2020a; Zeng *et al.*, 2020; Dai *et al.*, 2021; Shi *et al.*, 2021; Li *et al.*, 2022; Liu *et al.*, 2022). In the Yangtze River Delta, increased PM_10_, PM_2.5_, SO_2_, and CO before oocyte retrieval correlated with a reduced likelihood of live birth (Zhang *et al.*, 2022). A US study associated elevated odds of IVF failures with NO_2_ and PM_2.5_ increases (Gaskins *et al.*, 2019). Chengdu and Hebei identified a positive correlation between O_3_ levels and clinical pregnancy rates (Li *et al.*, 2020a; Zeng *et al.*, 2020), while other Chinese studies have not shown this (Qiu *et al.*, 2019; Dai *et al.*, 2021; Shi *et al.*, 2021; Liu *et al.*, 2022). Similar findings in a US study (Boulet *et al.*, 2019) revealed a positive O_3_ correlation with implantation rates, contrasting with studies in South Korea (Choe *et al.*, 2018), Italy (Iodice *et al.*, 2021), and Israel (Farhi *et al.*, 2014), which reported no such correlation. These diverse results underscore the complex relationship between air pollution and IVF/ICSI-ET outcomes, highlighting the need for further investigation and consideration of regional variations.

Notably, the distinct chemical compositions of PM_2.5_ and PM_10_ can elicit diverse biological and physiological responses in individuals exposed to these particles (Chen *et al.*, 2020; Bae *et al.*, 2022; Wang *et al.*, 2023a; Xiao *et al.*, 2023). Acknowledging the potential impact of environmental factors on reproductive outcomes in the context of IVF/ICSI-ET, the unique characteristics of PM_2.5_ and PM_10_ may lead to distinct impacts on fertility and pregnancy outcomes. Moreover, most existing studies have focused solely on studying a single pollutant (Boulet *et al.*, 2019; Qiu *et al.*, 2019; Dai *et al.*, 2021; Iodice *et al.*, 2021; Shi *et al.*, 2021; Liu *et al.*, 2022). Few investigations, to our knowledge (Fang *et al.*, 2023), have explored the combined impact of exposure to multiple air pollutants on pregnancy outcomes in women undergoing IVF/ICSI-ET. In reality, these women frequently encounter simultaneous exposure to diverse air pollutants (Wen *et al.*, 2023).

Therefore, our retrospective cohort study aims to investigate the association between exposure to mixed air pollutants and clinical pregnancy rates during various specific exposure periods of fresh IVF/ICSI-ET cycles in the Sichuan Basin of Southwest China, a region housing ∼100 million people and recognized as one of the most polluted areas (Gao *et al.*, 2018; Zhao *et al.*, 2018). We employ the Quantile g-computation (QG-C) method and Bayesian kernel machine regression (BKMR) method, innovative tools for assessing the overall impact of environmental pollutant mixtures on health (Bobb *et al.*, 2015; Keil *et al.*, 2020; Chen *et al.*, 2023; Wen *et al.*, 2023; Zhan *et al.*, 2023). These methods enable us to estimate the joint effects of air pollutant mixtures, providing a more comprehensive understanding of how combined exposure influences clinical pregnancy rates. This approach addresses a gap in existing research, which has primarily focused on individual pollutants, and highlights the unique strength of our study in examining the collective impact of multiple pollutants.

## Materials and methods

### Study design and patients

**Figure 1.**
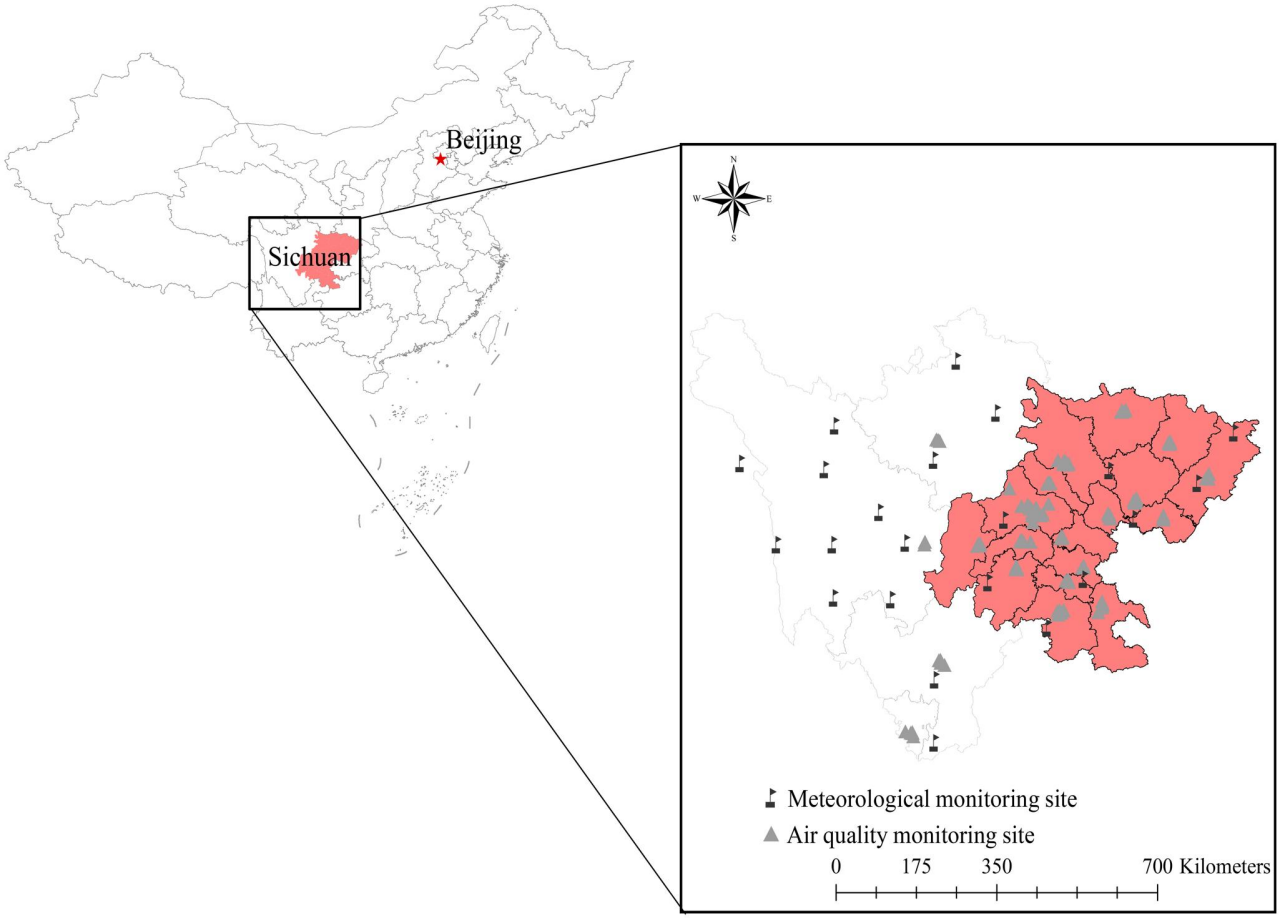
**Distribution of air quality monitoring stations and meteorological monitoring stations in 17 cities (red areas) in the Sichuan Basin of China**.

This retrospective cohort study involved infertile patients residing in the 17 cities of the Sichuan Basin who underwent their initial IVF/ICSI-ET cycle at the Centre for Assisted Reproduction of Sichuan Jinxin Xinan Women & Children’s Hospital between January 2020 and January 2023. Exclusions were made for patients meeting any of the following specific criteria: no fresh ET, incomplete clinical and address information, residency outside the 17 cities within the Sichuan Basin, age over 45 years, use of donor semen, thin endometrium (<8 mm), and infertility factors not related to tubal or ovulatory issues. Supplementary Fig. S1 shows the patient inclusion and exclusion process, while Fig. 1 delineates the study area.

### Ethical approval

The study received approval from the Ethics Committee of Sichuan Jinxin Xinan Women & Children’s Hospital (No. 2021014) and the Ethics Committee of Chongqing Medical University (No. 2021060).

### IVF/ICSI-ET procedures

In our study, all women underwent IVF/ICSI-ET following our center’s standard protocol, which consisted of six main steps: routine diagnosis of infertility in couples, ovarian stimulation, oocyte retrieval, IVF/ICSI, ET, and hCG testing for pregnancy (Ruan *et al.*, 2023; Wan *et al.*, 2023; Li *et al.*, 2024; Wang *et al.*, 2024).

### Ovarian stimulation protocol

Women in the study were allocated to various ovarian stimulation protocols based on their characteristics. In the follicular phase GnRH agonist protocol, women received a 3.75 mg injection of GnRH agonist (Beaufour Ipsen, Paris, France) on Days 2–5 of their menstrual cycle. Down-regulation (defined as follicle diameter ≤5 mm, estradiol (E2) <50 pg/ml, LH <5 mIU/ml) was confirmed after 28–37 days. For the luteal phase of the GnRH agonist protocol, a 0.1 mg dose of short-acting GnRH agonist (Ferring GmbH, North Rhine-Westphalia, Germany) was administered subcutaneously from the mid-luteal phase for 14–18 days until pituitary down-regulation was achieved (follicle diameter ≤5 mm, E2 < 50 pg/ml, LH < 5 mIU/ml). Subsequently, gonadotropin (Gn) (Merck Serono, Geneva, Switzerland) was administered with an initial dose of 100–225 IU until follicular maturation. In the GnRH antagonist protocol, Gn (Merck Serono) was generally administered at 100–300 IU per day from the 2nd to the 4th day of the menstrual cycle. A 0.25 mg dose of GnRH antagonist (Aeterna Zentaris, Quebec, Canada) was injected subcutaneously daily when the dominant follicle diameter was ≥ 12–14 mm or from the 5–6th day of administration until the trigger day. For the progestin-primed ovarian stimulation, oral medroxyprogesterone acetate (Xianju Pharmaceutical, Zhejiang, China) was taken at 6–10 mg/day from the 2nd to the 5th day of the menstrual cycle. Additionally, HMG (Lizhu Pharmaceutical, Guangdong, China) at 150–300 IU per day was administered concurrently.

### Oocyte retrieval, sperm preparation, and insemination

hCG (Lizhu Pharmaceutical, Guangdong, China) and/or GnRH agonist (Ferring Pharmaceuticals, Saint-Prex, Switzerland) injection was used as a trigger when at least three follicles reached ≥17 mm in diameter, followed by oocyte retrieval 34–36 h later.

Semen samples were collected from all participants through masturbation following a 3-day period of sexual abstinence. Post-collection, the samples were incubated at 37°C in an environment with 5% CO_2_ to facilitate liquefaction for a duration of 30 min. Standard semen parameter analysis was conducted according to the guidelines outlined in the WHO Laboratory Manual for the Examination and Processing of Human Semen (Cao *et al.*, 2011). Motile spermatozoa were isolated from the semen using the discontinuous density gradient method, which was effective for separating seminal plasma and other cellular components (Lu *et al.*, 2010).

Cumulus-enclosed oocytes were collected in 2.5 ml IVF medium (Vitrolife Sweden AB, Göteborg, Sweden) and kept in a 37°C incubator with 5% O_2_ and 6% CO_2_ for insemination. Insemination (IVF or ICSI) and embryo culture were conducted as previously described (Xiong *et al.*, 2011; Zhang *et al.*, 2013).

### ET and luteal support

Between the 3rd and 5th day post-fertilization, 1–2 grade I–II high-quality embryos were selectively transferred. The grading of embryos followed the guidelines of the Istanbul consensus (Alpha Scientists in Reproductive Medicine and ESHRE Special Interest Group of Embryology, 2011). Luteal phase support commenced on the day of oocyte retrieval using 200 mg vaginal progesterone (Besins Healthcare, Paris, France) administered every 8 h. Additionally, 20 mg of dydrogesterone (Abbott Laboratories, IL, USA) was taken twice daily. Serum β-hCG levels were monitored 14 days post-ET. If β-hCG was positive, the medications supporting the corpus luteum were continued without alteration. An ultrasound examination was performed 28 days post-ET. Upon confirmation of clinical pregnancy, the luteal phase support medications remained unchanged until 58 days post-ET.

### Outcome measure

The outcome measured was clinical pregnancy (binary), a pivotal indicator of the effectiveness of IVF/ICSI-ET therapy among the infertility population, reflecting the success of the procedure (1 = pregnancy, 0 = otherwise). Clinical pregnancy was defined as the ultrasound detection of an intrauterine gestational sac 5 weeks (35 days) after ET (Zhang *et al.*, 2022).

### Exposure assessment and exposure periods definition

We collected daily concentrations of ambient air pollutants including PM_2.5_, PM_10_, SO_2_, CO, NO_2_, and O_3_ (daily 8-h maximum moving-average level) from multiple monitoring stations situated across Sichuan Province. These data were collected from January 2019 to June 2023 and was sourced from the China Air Quality Online Monitoring and Analysis Platform. We also obtained the concurrent meteorological data from a Chinese scholar (https://quotsoft.net/air/) and the National Climatic Data Center, including daily temperature and dew point measurements. Employing inverse distance weighting (IDW), we spatially interpolated the daily air quality and meteorological data based on the latitude and longitude coordinates of each participant’s home address (Padula *et al.*, 2014; Padula *et al.*, 2019). The average ambient air pollutant concentration during the distinct periods of the IVF/ICSI-ET cycle was calculated (Supplementary Fig. S2): Period 1: average concentrations for the 90 days before oocyte retrieval, Period 2: average concentrations from oocyte retrieval to ET, Period 3: average concentrations from ET to the hCG test, and Period 4: average concentrations throughout the entire study period. The air quality checks in IVF laboratories adhere to the regulations of the Ministry of Health of China and undergo routine inspections and maintenance.

### Handling of missing data

Regarding missing clinical data, we divided our approach into two parts. Patients who were missing key variables, such as address and pregnancy status, were excluded from the study. Other variables, such as BMI and duration of infertility, with missing rates of <20% were imputed using random forest imputation. For pollutant data, raw data from Sichuan Province monitoring stations were processed. Stations with more than 20% air pollutants missing data were excluded, while for those with <20% missing data, the Last Observation Carried Forward (LOCF) method was applied.

### Statistical analysis

All continuous variables underwent a normality test, revealing a *P*-value <0.05, indicating their departure from a normal distribution. Descriptive statistics included medians (interquartile range, IQR) for continuous variables and numbers (%) for categorical variables. Ambient air pollutant concentrations during various exposure periods were summarized with mean, SD, range, median, and IQR. Spearman’s rank correlation coefficients (*r*_s_) were used to assess paired pollutant correlations across the four periods.

Logistic regression models were employed to evaluate the impact of exposure to a single air pollutant on clinical pregnancy rates in IVF/ICSI-ET patients. Covariates were selected based on: (i) variables recognized as potential risk factors for IVF/ICSI-ET pregnancy outcomes according to current knowledge (Chambers *et al.*, 2016) and (ii) variables included in previous epidemiologic studies on IVF/ICSI-ET outcomes (Shi *et al.*, 2021; Xue *et al.*, 2021; Zhang *et al.*, 2022), including maternal age, BMI, stimulation protocol, endometrial thickness on hCG day, total dose of Gn, duration of infertility, cause of infertility, infertility type, number of retrieved oocytes, number of ETs, stage of ET, temperature, and dew point. It was worth noting that before finalizing our model, we performed a multicollinearity test to check for high correlations between variables, the occurrence of which may affect the accuracy of the model. To this end, we calculated the variance inflation factor (VIF). A VIF of <4 would indicate minimal correlation between the variables, thus allowing us to proceed with the multivariate logistic regression model with confidence (Wu *et al.*, 2023). This rigorous process ensured that our model was reliable and produced accurate results.

The QG-C and BKMR methods were employed to evaluate the collective effects of exposure to multiple air pollutants on clinical pregnancy rates in IVF/ICSI-ET patients. QG-C analyzed all air pollutants as a composite to assess their combined influence on clinical pregnancy (Keil *et al.*, 2020). Additionally, the BKMR approach was applied to ascertain the reliability of the QG-C model and explore potential interactions between air pollutants (Bobb *et al.*, 2015). In the QG-C model, the combined impact of the air pollutants mixture on clinical pregnancy rates was assessed using the coefficient ψ, which represented the logarithm of the odds ratio (OR). Precisely, it delineated the effect on study outcome when the concentration of each pollutant was concurrently increased by one quartile. A positive ψ implied a positive correlation, signifying that an escalation in pollutant concentration aligns with a higher likelihood of clinical pregnancy rates. Conversely, a negative ψ indicated an inverse relationship. Additionally, in the BKMR model, ‘Estimated’ indicated the estimated effect or coefficient of mixed contamination associated with the likelihood of clinical pregnancy. When all ambient air pollutants were higher than their 50th percentile, the estimated results were considered statistically significant if the 95% CI did not contain zero. When the estimate was negative, it indicated that there was an inverse relationship between mixed contamination and the likelihood of clinical pregnancy rates. In other words, higher levels of mixed contamination were associated with a decreased likelihood of clinical pregnancy. Conversely, if the estimate was positive, it suggested a positive relationship, meaning higher levels of mixed contamination were associated with an increased likelihood of clinical pregnancy rates. This comprehensive approach strengthened the study’s findings and provided a clearer understanding of the associations between air pollution and clinical pregnancy.

Additionally, we conducted multiple sensitivity analyses to validate the reliability of our study findings. (i) We used a dual-pollutant model to assess the independent associations between each pollutant and clinical pregnancy rates in IVF/ICSI-ET patients. (ii) A previous study (Li *et al.*, 2018) had indicated a correlation between pollutants and ovulatory disorders. To mitigate the confounding effect of ovulatory dysfunction, we excluded these patients and focused exclusively on those with tubal infertility for our analysis. (iii) We also explored the impact of temporal trends on our study results. Specifically, we included the year of ET as a new adjusting variable in our QG-C model and reanalyzed the data. (iv) We conducted reanalysis using Distributed Lag Nonlinear Models (DLNM) to explore the influence of exposure during other time periods on our results, leveraging its capability to automatically correct for effects across various time windows. Stratified analyses examined the effects of air pollutants by age groups (20–29 years, 29–35 years, and 36–45 years), BMI categories (<18.5 kg/m^2^, 18.5–24 kg/m^2^, and ≥24 kg/m^2^), and the number of embryos transferred (one vs two).

Adjusted ORs and 95% CIs for clinical pregnancy rates during different exposure periods were reported in association with each IQR increment of ambient air pollutants. All analyses were conducted using R software (Version 4.3.1, Vienna, Austria), and statistical significance was set at *P-*value <0.05 based on two-tailed calculations.

## Results

### Participants’ characteristics and ambient air pollutant exposure

In our study, we included a total of 34 453 female patients who underwent infertility diagnosis and subsequently proceeded with ovarian stimulation procedures. Following inclusion and exclusion criteria, this study included 5208 women undergoing their initial fresh IVF/ICSI-ET (Supplementary Fig. S1). Notably, all patients were from the 17 cities within the Sichuan Basin in China (Fig. 1). Table 1 details the characteristics of participants, with an average age and BMI of 31.00 (28.00, 34.00) years and 21.77 (19.95, 24.03) kg/m^2^, respectively. Among them, 33.30% opted for single ET, while 66.70% chose double ET. Additionally, 62% underwent cleavage-stage ET and the remaining 38% had blastocyst stage transfers. The clinical pregnancy rate for participants during the study period stood at 54.53%.

**Table 1.** Characteristics of 5208 participants at baseline.

Variables	All (N = 5208)	Non-pregnancy (N = 2368)	Pregnancy (N = 2840)	*P* -value
Age (years)^a^	31.00 (28.00, 34.00)	31.00 (28.00, 34.00)	31.00 (28.00, 33.00)	<0.001
BMI (kg/m^2^)^a^	21.77 (19.95, 24.03)	21.52 (19.81, 23.87)	21.88 (20.03, 24.14)	0.003
Endometrial thickness on hCG day (mm)^a^	10.50 (9.50, 12.00)	10.50 (9.50, 12.00)	10.50 (9.50, 12.00)	0.001
Total dose of Gn (IU)^a^	1950.00 (1575.00, 2475.00)	1950.00 (1575.00, 2475.00)	2000.00 (1575.00, 2475.00)	0.571
Duration of infertility (years)^a^	3.00 (1.00, 4.00)	3.00 (1.00, 5.00)	3.00 (1.00, 4.00)	0.144
Number of retrieved oocytes^a^	10.00 (7.00, 13.00)	9.00 (6.00, 13.00)	10.00 (7.00, 13.00)	<0.001
Number of embryo transfer, n (%)				
Single	1735 (33.30)	829 (35.00)	906 (31.90)	0.019
Double	3473 (66.70)	1539 (65.00)	1934 (68.10)	
Stage of embryo transfer, n (%)				
Cleavage	3230 (62.00)	1525 (64.40)	1705 (60.00)	0.001
Blastocyst	1978 (38.00)	843 (35.60)	1135 (40.00)	
Stimulation protocol, n (%)				
GnRH antagonist	2321 (44.60)	1101 (46.50)	1220 (43.00)	0.036
GnRH agonist	2839 (54.50)	1247 (52.70)	1592 (56.10)	
Other protocols	48 (0.90)	20 (0.80)	28 (1.00)	
Infertility type, n (%)				
Primary infertility	2471 (47.40)	1123 (47.40)	1348 (47.50)	0.999
Secondary infertility	2737 (52.60)	1245 (52.60)	1492 (52.50)	
Cause of infertility, n (%)				
Tubal factor	4761 (91.40)	2153 (90.90)	2608 (91.80)	0.263
Ovulation disorders	447 (8.60)	215 (9.10)	232 (8.20)	
Year of embryo transfer, n (%)				
2020	1600 (30.70)	714 (30.20)	886 (31.20)	0.365
2021	1798 (34.50)	837 (35.30)	961 (33.80)	
2022	1640 (31.50)	732 (30.90)	908 (32.00)	
2023	170 (3.30)	85 (3.60)	85 (3.00)	

Abbreviations: Gn, gonadotropin.

a Cited as medians (interquartile range, IQR).

Average daily concentrations of six ambient air pollutants and meteorological variables during different exposure periods are presented in Supplementary Tables S1 and S2. The definition of the periods is shown in Supplementary Fig. S2. Throughout the study periods, maternal averaged concentrations were 36.65 µg/m^3^ (PM_2.5_), 57.26 µg/m^3^ (PM_10_), 6.51 µg/m^3^ (SO_2_), 29.30 µg/m^3^ (NO_2_), 0.66 mg/m^3^ (CO), and 87.77 µg/m^3^ (O_3_), respectively, with average temperatures of 16.36°C and dew points of 0.46°C. Supplementary Fig. S3 illustrates *r_s_* for pollutants during each exposure period, revealing a high correlation between PM_10_ and PM_2.5_ (*r_s_* > 0.90, *P*-value < 0.05).

### Relationship between exposure to ambient air pollution and clinical pregnancy rates during specific exposure periods in the single-pollutant models

The ORs and their corresponding 95% CIs for the likelihood of clinical pregnancy per IQR increase in six air pollutants (PM_2.5_, PM_10_, SO_2_, NO_2,_ CO, and O_3_) during each exposure period are detailed in Table 2. Two multivariate logistic regression models (partially adjusted model and fully adjusted model) were developed. Subsequently, multicollinearity diagnosis was conducted, revealing that the VIF of the variables in both models was <4, indicating that no variables were eliminated from the models. In the partially adjusted model, the estimated ORs for the likelihood of clinical pregnancy were as follows: 0.88 (95% CI: 0.81–0.96) for PM_2.5_ in Periods 1 and 4, 0.90 (95% CI: 0.82–0.98) for PM_10_ in Periods 1 and 4, 0.91 (95% CI: 0.85–0.98) and 0.93 (95% CI: 0.87–0.99) for SO_2_ in Periods 3 and 4, 0.92 (95% CI: 0.86–0.98) and 0.93 (95% CI: 0.87–0.99) for CO in Periods 1 and 4, and 1.17 (95% CI: 1.07–1.28), 1.10 (95% CI: 1.01–1.19), and 1.16 (95% CI: 1.06–1.28) for O_3_ in Periods 1, 2, and 4, respectively. In the partially adjusted model, the majority of pollutants displayed associations with the likelihood of clinical pregnancy. In the fully adjusted model, temperature and dew point were further adjusted based on the partially adjusted model. Even after this extra adjustment, statistically significant associations of PM_2.5_, SO_2_, and O_3_ with the possibility of clinical pregnancy persisted. The estimated ORs were 0.83 (95% CI: 0.70–0.99) and 0.83 (95% CI: 0.69–0.98) for PM_2.5_ in Periods 1 and 4, 0.92 (95% CI: 0.86–0.99) for SO_2_ in Period 3, and 1.17 (95% CI: 1.01–1.36) for O_3_ in Period 1.

**Table 2.** Association between ambient air pollutant exposure and clinical pregnancy rates during specific exposure periods in the single pollutant models.

	Ambient air pollution	Period 1		Period 2		Period 3		Period 4	
		aOR (95% CI)	*P* -value	aOR (95% CI)	*P* -value	aOR (95% CI)	*P* -value	aOR (95% CI)	*P* -value
Partially adjusted model^a^	PM_2.5_	**0.88 (0.81–0.96)**	0.003	0.96 (0.89–1.03)	0.229	0.93 (0.86–1.01)	0.075	**0.88 (0.81–0.96)**	0.003
	PM_10_	**0.90 (0.82–0.98)**	0.017	0.97 (0.90–1.05)	0.516	0.93 (0.85–1.01)	0.065	**0.90 (0.82–0.98)**	0.014
	SO_2_	0.94 (0.87–1.01)	0.070	0.98 (0.91–1.05)	0.497	**0.91 (0.85–0.98)**	0.010	**0.93 (0.87–0.99)**	0.047
	NO_2_	0.93 (0.86–1.01)	0.106	0.99 (0.91–1.06)	0.710	0.94 (0.87–1.01)	0.095	0.93 (0.86–1.01)	0.090
	CO	**0.92 (0.86–0.98)**	0.016	1.01 (0.94–1.08)	0.805	0.98 (0.92–1.05)	0.540	**0.93 (0.87–0.99)**	0.031
	O_3_	**1.17 (1.07–1.28)**	<0.001	**1.10 (1.01–1.19)**	0.026	1.06 (0.97–1.15)	0.222	**1.16 (1.06–1.28)**	0.001
Fully adjusted model^b^	PM_2.5_	**0.83 (0.70–0.99)**	0.036	0.99 (0.90–1.09)	0.825	0.96 (0.85–1.07)	0.441	**0.83 (0.69–0.98)**	0.032
	PM_10_	0.91 (0.78–1.07)	0.263	1.01 (0.92–1.11)	0.834	0.95 (0.85–1.05)	0.303	0.90 (0.77–1.06)	0.198
	SO_2_	0.96 (0.89–1.03)	0.276	0.98 (0.91–1.05)	0.492	**0.92 (0.86–0.99)**	0.020	0.96 (0.89–1.03)	0.234
	NO_2_	0.99 (0.90–1.09)	0.835	1.02 (0.94–1.11)	0.650	0.96 (0.88–1.05)	0.383	0.98 (0.89–1.09)	0.726
	CO	0.96 (0.87–1.06)	0.389	1.06 (0.98–1.14)	0.177	1.03 (0.94–1.12)	0.527	0.98 (0.88–1.08)	0.667
	O_3_	**1.17 (1.01–1.36)**	0.032	0.99 (0.88–1.12)	0.924	0.96 (0.83–1.10)	0.566	1.14 (0.99–1.32)	0.073

Abbreviations: Period 1, 90 days before oocyte retrieval; Period 2, oocyte retrieval to embryo transfer; Period 3, embryo transfer to serum hCG test; Period 4, 90 days before oocyte retrieval to serum hCG test; aOR, adjusted odds ratio; PM_2.5_, fine particulate matter, PM_10_, inhalable PM; CO, carbon monoxide; NO_2_, nitrogen dioxide; O_3_, ozone; SO_2_, sulfur dioxide; Gn, gonadotropin.

The numbers in bold indicate a statistically significant association.

a Adjusted for age, BMI, stimulation protocol, endometrial thickness on hCG day, total dose of Gn, duration of infertility, cause of infertility, infertility type, number of retrieved oocytes, number of embryo transfer, and stage of embryo transfer.

b Further adjusted for meteorological factors, including temperature and dew point.

### Association between exposure mixture pollutant and clinical pregnancy rates during specific exposure periods in the multi-pollutant models

**Figure 2.**
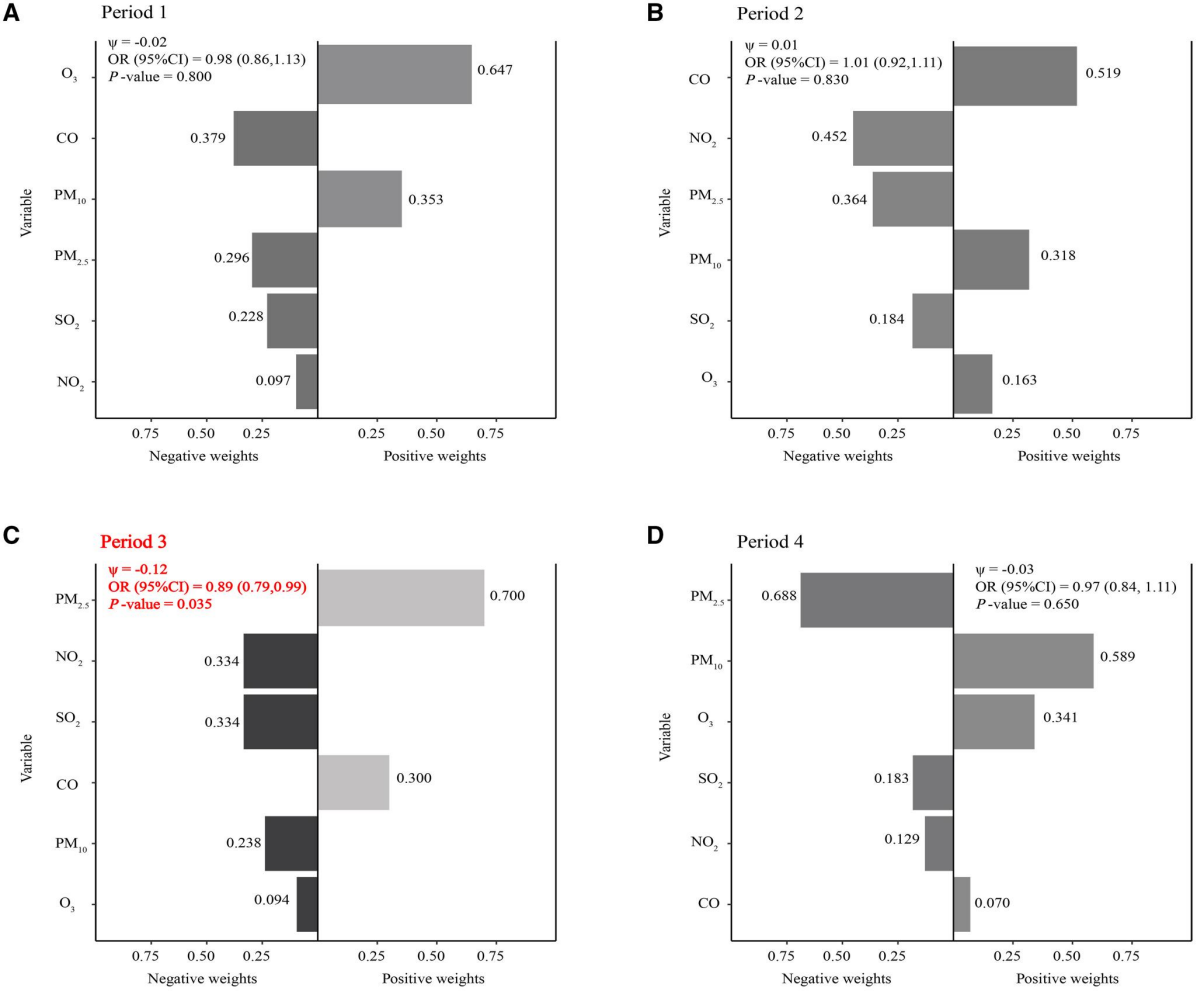
**Quantile G-Computation (QG-C) regression analysis of mixture air pollution exposure’s impact on clinical pregnancy likelihood across various exposure periods**. Using the QG-C model, after adjusting for age, BMI, stimulation protocol, endometrial thickness on hCG day, total dose of Gn, duration of infertility, cause of infertility, infertility type, number of retrieved oocytes, number of embryo transfer, stage of embryo transfer, temperature, and dew point, the relationship between the weights of each pollutant and the likelihood of clinical pregnancy was assessed across four distinct time periods: Period 1 (**A**), Period 2 (**B**), Period 3 (**C**), Period 4 (**D**). Period 1, 90 days before oocyte retrieval; Period 2, oocyte retrieval to embryo transfer; Period 3, embryo transfer to serum hCG test; Period 4, 90 days before oocyte retrieval to serum hCG test; Gn, gonadotropin; PM_2.5_, fine particulate matter (particles ≤ 2.5 µm); PM_10_, inhalable particulate matter (particles ≤ 10 µm); CO, carbon monoxide; NO_2_, nitrogen dioxide; O_3_, ozone; SO_2_, sulfur dioxide; ψ, logarithm of the odds ratio; OR, odds ratio.

**Figure 3.**
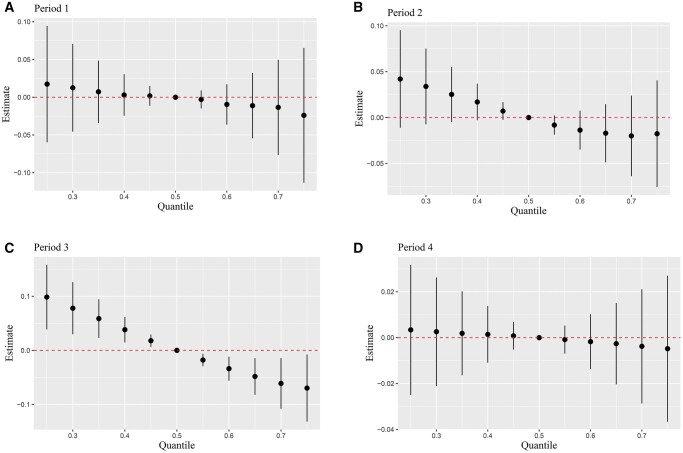
**Bayesian kernel machine regression (BKMR) analysis of mixture air pollution exposure’s impact on clinical pregnancy likelihood across various exposure periods**. The cumulative effects of air pollution mixtures on clinical pregnancy likelihood across four exposure periods evaluated by the BKMR models: Period 1 (**A**), Period 2 (**B**), Period 3 (**C**), Period 4 (**D**). The model was adjusted for variables including age, BMI, stimulation protocol, endometrial thickness on hCG day, total dose of Gn, duration of infertility, cause of infertility, infertility type, number of retrieved oocytes, number of embryo transfer, stage of embryo transfer, temperature, and dew point. Period 1, 90 days before oocyte retrieval; Period 2, oocyte retrieval to embryo transfer; Period 3, embryo transfer to serum hCG test; Period 4, 90 days before oocyte retrieval to serum hCG test; Gn, gonadotropin.

Figures 2 and 3 show the results of multi-pollutant models based on QG-C and BKMR methods. In Fig. 2C, the QG-C model revealed a statistically significant 12% reduction in the likelihood of clinical pregnancy associated with the combined effect of ambient air pollutants in Period 3 (*P*-value = 0.035). In the negative association effect, NO_2_ (33.40%) and SO_2_ (33.40%) were the primary driving factors, followed by PM_10_ (23.80%) and O_3_ (9.40%). Figure 3 shows the combined effect of ambient air pollutants on clinical pregnancy estimated from BKMR for each exposure period. Our study demonstrated that as pollutant concentrations increased concurrently, the likelihood of clinical pregnancy decreased across all exposure periods. However, a distinct trend toward a decrease in the probability of clinical pregnancy was observed only in exposure Period 3, when the six pollutants reached or exceeded their 50th percentile (*P*-value < 0.05). This underscored a significant combined effect of ambient air pollution during this period on clinical pregnancy. Both BKMR and QG-C analyses concurred that elevated mixed pollutants in Period 3 were correlated with a decreased possibility of clinical pregnancy, confirming the robustness of our findings. In addition, the interaction between ambient air pollutants was explored using the BKMR, as illustrated in Supplementary Fig. S4. The study findings indicated an interaction between O_3_ and SO_2_ during Period 2.

### Sensitivity analysis and stratified analysis

**Figure 4.**
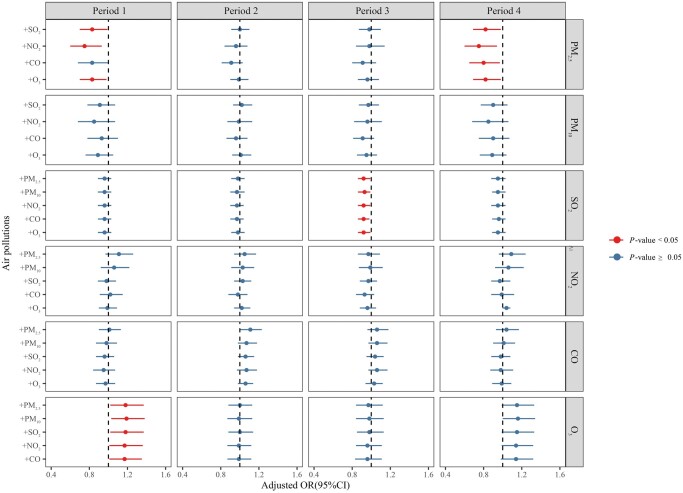
**Associations between air pollutant exposure and clinical pregnancy likelihood at each period: two-pollutant model results**. The logistic regression models were adjusted for a range of factors including age, BMI, stimulation protocol, endometrial thickness on the day of hCG administration, total gonadotropin dose, duration and cause of infertility, infertility type, number of retrieved oocytes, number of embryos transferred, stage of embryo transfer, temperature, and dew point. Adjusted pollutants are depicted on the far left, while the main pollutants are on the far right. Odds ratios (ORs) and their corresponding 95% CIs are represented by circles and error bars, with significant associations at the *P*-value < 0.05 indicated in red. Period 1, 90 days before oocyte retrieval; Period 2, oocyte retrieval to embryo transfer; Period 3, embryo transfer to serum hCG test; Period 4, 90 days before oocyte retrieval to serum hCG test; Gn, gonadotropin; PM_2.5_, fine particulate matter (particles ≤ 2.5 µm); PM_10_, inhalable particulate matter (particles ≤ 10 µm); CO, carbon monoxide; NO_2_, nitrogen dioxide; O_3_, ozone; SO_2_, sulfur dioxide.

To affirm the reliability of the single-pollution model, a two-pollutants analysis was performed (Fig. 4). However, when both PM_2.5_ and PM_10_ were included, the VIF exceeded 4. Consequently, in the dual-pollution model, they were not allowed to be included together. Notably, this model revealed inverse correlations between PM_2.5_ in Periods 1 and 4, SO_2_ in Period 3, and the occurrence of clinical pregnancy, corroborating the results of the fully adjusted logistics model. Additionally, O_3_ in Period 1 exhibited a protective effect on clinical pregnancy after adjusting for other pollutants. It could be seen that the result of our single pollution model was reliable.

After excluding patients with ovulatory disorders and focusing solely on those with tubal issues (Supplementary Fig. S5), we found results consistent with the primary QG-C analysis (Fig. 2). The QG-C model (Supplementary Fig. S5C) demonstrated that the combined effect of environmental air pollutants during the Period 3 was associated with a 13% decrease in the likelihood of clinical pregnancy (*P*-value = 0.027), which was statistically significant. In terms of negative effects, SO_2_ (37.50%) and NO_2_ (27.50%) were the primary driving factors, followed by PM_10_ (19.10%) and O_3_ (15.90%). Supplementary Fig. S6 shows the impact of time trends on the study outcome. According to the QG-C model, the combined effect of environmental air pollutants during Period 3 was associated with a 12% reduction in the likelihood of clinical pregnancy (*P*-value = 0.037), which was statistically significant. In terms of negative effects, NO_2_ (39.50%) and SO_2_ (35.10%) were identified as the primary driving factors, followed by PM_10_ (18.70%) and O_3_ (6.80%). DLNM was utilized to adjust for other exposure periods (Supplementary Fig. S7), revealing that the negative impact of SO_2_ exposure during Period 3 on clinical pregnancy persisted even after adjusting for other exposure windows.

In the stratified analysis, dividing the patients into groups based on the number of embryos transferred (one vs two), the results showed that those who received two embryos were more susceptible to the negative effects of SO_2_ and mixed pollutants during Period 3 (Supplementary Figs S8, S9, and S10). Furthermore, BMI-based grouping into lean, normal, and obese categories indicated increased vulnerability to infertility in the obese group (BMI ≥ 24 kg/m^2^) when exposed to PM_2.5_ and PM_10_ in Period 1 (Supplementary Fig. S11). Finally, age groups (20–29 years, 30–35 years, and 36–45 years) revealed heightened susceptibility of infertility patients aged 20–29 years to PM_2.5_ in Periods 1 and 4, resulting in a diminished probability of clinical pregnancy (Supplementary Fig. S12).

## Discussion

Monitoring clinical pregnancy rates provides valuable insights into the effectiveness of treatment and contributes to the ongoing improvement of IVF/ICSI-ET protocols and procedures. In this study, which included 5208 patients undergoing their initial fresh IVF/ICSI-ET cycle across 17 cities in the Sichuan Basin of China, we explored the correlation between exposure to single and/or combined ambient air pollutants and clinical pregnancy rates at various periods during fresh IVF/ICSI-ET cycles. Our findings underscored the heightened sensitivity observed during Period 3 (from ET to the serum hCG test). Exposure to SO_2_ or a combination of air pollutants was associated with a decreased likelihood of clinical pregnancy during this specific period. Stratified analyses further underscored the vulnerability to ambient air pollution of individuals who received two embryos, those with a BMI ≥24 kg/m^2^, or those under age 35, particularly in the 20–29 age group, with a specific focus on PM_2.5_ and SO_2_.

The impact of air pollutants on ART outcomes had been predominantly explored through single-pollutant models. Notably, studies had identified a connection between O_3_ and diminished ovarian reserve in women (Liu *et al.*, 2024), which was associated with significant declines in clinical pregnancy rates, live births, and single live births in IVF (Hu *et al.*, 2020b). The surge in O_3_ concentrations in China, attributed to long-term climate changes and increased emissions of anthropogenic O_3_ precursors (Zhang *et al.*, 2019), poses a pressing public health concern (Li *et al.*, 2019a). This underscored the substantial influence of meteorological conditions on the frequency and duration of ambient air pollution events (Ma *et al.*, 2023). Despite this, many existing studies inadequately accounted for the impact of meteorological factors on pregnancy outcomes. In our study, even after adjusting for meteorological factors, O_3_ continued to exhibit a protective effect on clinical pregnancy, consistent with recent research (Boulet *et al.*, 2019; Zeng *et al.*, 2020; Zhang *et al.*, 2022). However, it was crucial to acknowledge that some studies had reported a negative correlation between O_3_ and clinical pregnancy (Wu *et al.*, 2021) as well as live birth (Shi *et al.*, 2021). These variations might be attributed to diverse population characteristics, exposure estimation methods, and exposure periods. Therefore, further research and comprehensive analysis should consider these multifaceted factors to enhance our understanding of how O_3_ affects women’s reproductive health.

Turning to particulate matter, a significant air quality pollutant, our study unveiled a consistent association between increased PM_2.5_ concentration and adverse pregnancy outcomes. This aligned with findings in both naturally conceived populations (Li *et al.*, 2019b; Liang *et al.*, 2019; Zhao *et al.*, 2021) and assisted reproductive populations of other studies (Wang *et al.*, 2019; Jin *et al.*, 2022; Liu *et al.*, 2022; Zhang *et al.*, 2022; Wang *et al.*, 2023b). Importantly, our investigation revealed that PM_2.5_ exposure exerted an impact on clinical pregnancy rates, particularly during exposure Periods 1 (90 days) and 4 (108 days), hinting at potential adverse consequences linked to extended systemic exposure. This underscored the significance of exploring the mechanisms between PM_2.5_ and assisted reproductive treatment outcomes for enhanced treatment efficacy and patient wellbeing.

Furthermore, our study identified the association between SO_2_ exposure and clinical pregnancy rates during Period 3. To bolster the credibility of our findings, a dual pollutant analysis was conducted, yielding results consistent with the single-pollution model. This consistent pattern enhanced the stability of our results. In addition, two other Chinese studies (Zeng *et al.*, 2020; Liu *et al.*, 2022) concurred on the detrimental impact of SO_2_ exposure during this period on clinical pregnancy. However, beyond the common finding related to SO_2_, one study revealed negative effects of exposure to other pollutants (PM_2.5_, NO_2_, and CO) during this period on clinical pregnancy and biochemical pregnancy. Another study also reported adverse effects of PM_10_ exposure on clinical pregnancy and live births. Similarly, a South Korean study (Choe *et al.*, 2018) did not observe an impact of SO_2_ exposure during this period on pregnancy outcomes, but did identify that exposure to PM_10_ and NO_2_ during this phase was associated with a diminished likelihood of intrauterine pregnancy and biochemical pregnancy loss. Differences in the study populations might have contributed to these results. Nonetheless, we can still propose a hypothesis that exposure to air pollutants during this specific stage (Period 3) might influence embryo-to-endometrial interactions, thereby negatively affecting the endometrial lining’s implantation process. In future studies, more research was needed to confirm our opinion.

To date, research on the impact of ambient air pollutants on ART outcomes had predominantly relied on single-pollutant models, as evident in prior studies. Despite the real-world scenario involving simultaneous exposure to a mixture of pollutants (Wen *et al.*, 2023), few studies had delved into the combined effects of ambient air pollutants on this cohort. Consequently, there was a critical need to investigate the combined impact of ambient air pollutants on pregnancy outcomes in IVF/ICSI-ET patients. In this field, various methodologies, including weighted quantile sum (WQS) regression (Carrico *et al.*, 2015), BKMR (Bobb *et al.*, 2015), and QG-C (Keil *et al.*, 2020), were employed. WQS regression, a prevalent model in environmental epidemiology, constructs a weighted index to assess cumulative environmental chemical influences on outcomes (Carrico *et al.*, 2015; Chen *et al.*, 2022; Guo *et al.*, 2022). However, a significant limitation of the WQS regression is that it cannot simultaneously evaluate the combined effects of chemicals with different directions of action. QG-C, an innovative analytical method, extends the capabilities of WQS, eliminating the necessity for linear and additive effects (Keil *et al.*, 2020), and can concurrently assess the combined effects of chemicals with varying directions of action. BKMR addresses the potential non-linearity and non-additivity of variables (Bobb *et al.*, 2015), and has been widely used to study health problems potentially caused by exposure to various chemical contaminants (Guo *et al.*, 2016). BKMR utilizes a Markov chain Monte Carlo algorithm to filter variables and construct Gaussian kernel functions (Bobb *et al.*, 2015). The Gaussian kernel function can flexibly model complex relationships between response variables and multiple predictive variables, allowing for the visualization of potential interactions among these predictive variables.

Considering the advantages and limitations of the above models, our study opted for QG-C and BKMR to scrutinize the combined effects of ambient air pollution on clinical pregnancy. In analyzing mixed pollutants, QG-C observed that each IQR increase in the combination of air pollutants during Period 3 was associated with a decreased likelihood of clinical pregnancy. In the context of negative associations, NO_2_ (33.40%) and SO_2_ (33.40%) were identified as the main driving factors. This indicated that exposure to these pollutants significantly reduced the likelihood of clinical pregnancy. The high contributions of NO_2_ and SO_2_ underscored their substantial impact on reproductive outcomes. As mentioned earlier, we found that isolated exposure to SO_2_ during the third period leads to a decrease in clinical pregnancy rates. Now, in the exposure to a mixture during Period 3, SO_2_ again plays a crucial role, consistent with both sets of results. Regarding why no significant effect of NO_2_ exposure alone during the Period 3 was observed, it might be due to the nonlinear effect of NO_2_ during this period, making it difficult for the logistic regression model (which captures only linear relationships) to clearly detect this association. BKMR and QG-C results aligned accordingly.

Furthermore, an interaction between O_3_ and SO_2_ during Period 2 were found using BKMR. The interaction between O_3_ and SO_2_ during this period suggested that these pollutants may not act independently but rather interact with each other. For instance, the presence of SO_2_ could modify the effect of O_3_ exposure, potentially amplifying or mitigating its impact on pregnancy success rates. The identified interactions raise more public health concerns and provide a valuable avenue for future research. However, since this study primarily focused on the impact of mixed pollution exposure on clinical pregnancy, we only made a preliminary observation of this interaction and did not conduct an in-depth investigation. This finding indicates the need for more focused research to explore this interaction further in the future. Hence, identifying Period 3 as the pivotal sensitive period, our study found that exposure to a single pollutant, double pollutants, or a mixture of pollutants during this period demonstrated a negative association with the likelihood of clinical pregnancy as ambient air pollution concentrations increased. Both models unveiled a detrimental combined effect during Period 3, accentuating the pivotal role of this exposure phase in future treatments. However, further independent studies were essential to substantiate these observed combined effects.

Women undergoing IVF/ICSI-ET could pinpoint a precise exposure window since most crucial events in the IVF/ICSI-ET cycle were scheduled by caregivers. Similar to existing studies (Choe *et al.*, 2018; Boulet *et al.*, 2019; Zeng *et al.*, 2020; Wu *et al.*, 2021; Liu *et al.*, 2022), we selected four distinct exposure periods. Notably, Period 3 (ET to hCG test) emerged as the most sensitive, indicating a reduction in the likelihood of clinical pregnancy associated with air pollutant exposure. Consistent findings in previous studies highlighted the adverse effects of air pollutants during this period on biochemical pregnancy (Choe *et al.*, 2018), clinical pregnancy (Wu *et al.*, 2021; Liu *et al.*, 2022) and live birth (Boulet *et al.*, 2019; Wu *et al.*, 2021). These results underscored the significance of acknowledging and addressing air pollution during the ET to hCG test period to mitigate potential adverse impacts on clinical pregnancy rates.

Obesity in women might have significantly adverse effects on IVF/ICSI-ET pregnancy outcomes (Sermondade *et al.*, 2019). Our study indicated that BMI ≥ 24 kg/m^2^ patients were more susceptible to the adverse effects of ambient air pollutions (PM_2.5_ and PM_10_), consistent with a review highlighting increased risks in overweight or obese pregnant women exposed to ambient air pollution (Westergaard *et al.*, 2017). Furthermore, female age significantly influenced reproductive success, correlating inversely with oocyte production and mass (Igarashi *et al.*, 2015). As advanced maternal age was typically defined as 35 years or older at delivery (Reddy *et al.*, 2006), we categorized age into three groups (20–29, 30–35, and 36–45 years) for stratified analysis. Our findings revealed increased vulnerability to air pollutants, particularly among those younger than 35 years, emphasizing a consistent trend with existing literature (Zeng *et al.*, 2020; Shi *et al.*, 2021). However, in the age group over 35 years, the association between air pollutants and pregnancy outcomes was not distinctly evident. This might be attributed to reproductive aging-related factors, such as diminished oocyte quality and mitochondrial activity, chromosomal abnormalities, and heightened cell dysfunction, potentially overshadowing the impact of ambient air pollution on IVF/ICSI-ET outcomes (Cimadomo *et al.*, 2018). Transferring multiple embryos could increase pregnancy rates but also lead to a higher rate of multiple pregnancies, which could negatively affect obstetric and neonatal outcomes (Practice Committee of American Society for Reproductive Medicine, 2012). In our study, subjects had a maximum of two embryos transferred. We found that patients who received two embryos were more susceptible to the adverse effects of air pollution, a finding consistent with a study from China (Zhang *et al.*, 2022). This increased susceptibility might be due to the heightened physiological demands and increased sensitivity during the embryo implantation period.

There were several advantages to our study. Our study stood as a unique perspective on the environmental challenges faced by IVF/ICSI-ET patients within the distinctive region of the Sichuan Basin. This geographical context, renowned for its elevated pollution levels, had been insufficiently explored, and our research serves as a valuable contribution to understanding the dynamics of ambient air pollution in this specific locale. Unlike conventional studies, we have taken a divergent approach by intricately exploring the combined effects of exposure to multiple pollutants. This methodological innovation provided a nuanced and comprehensive understanding of the intricate relationship between ambient air pollution and clinical pregnancy rates, contributing to the depth of insights into the complexities of environmental impacts on assisted reproduction. Furthermore, the temporal sensitivity highlighted in our results, particularly during Period 3, illuminated a critical exposure period where the risk of reduced clinical pregnancy was elevated. Additionally, our stratified analyses revealed the vulnerability of specific populations, emphasizing the importance of considering individual characteristics, such as BMI and age, in understanding the effects of ambient air pollution, particularly PM_2.5_. Overall, our study enriched the scientific understanding of the complexities surrounding ambient air pollution and its impact on assisted reproduction outcomes, providing valuable insights for both researchers and clinicians.

This study had several limitations that warrant consideration. First, our retrospective design implied the results should be approached with caution, as retrospective studies were susceptible to selection bias due to non-random sampling of the population. Second, lifestyle factors such as smoking and alcohol consumption (Strak *et al.*, 2017), known confounders in IVF/ICSI-ET, were not accounted for, though their prevalence was generally low among women undergoing these procedures in China (Sansone *et al.*, 2015). Third, we only considered successful cycles that reached the hCG test, excluding the few patients who did not reach the IVF-ET or ICSI-ET stage. Fourth, we did not adjust for multiple comparisons, making our results exploratory and susceptible to type I errors (Li *et al.*, 2020b). Finally, we used the inverse distance weighting method to estimate pollutant concentrations at individual residential addresses but did not gather data on participants’ work locations or time–activity patterns, which could impact the accuracy of exposure predictions and should be considered when interpreting our findings.

## Conclusion

In summary, our study enriched the scientific understanding of the complexities surrounding ambient air pollution and its impact on ART outcomes. The insights gained hold relevance for both researchers and clinicians, offering valuable guidance in navigating the environmental challenges faced by IVF/ICSI-ET patients. As we contribute to the growing body of knowledge in this field, we hope our findings pave the way for further research and interventions aimed at mitigating the impact of ambient air pollution on assisted reproduction.

## Supplementary Material

hoae051_Supplementary_Data

## Data Availability

Request for access to the data should be made to the corresponding author at dingyb@cqmu.edu.cn. Data could be made available provided the applicant has appropriate ethics and author approval.
