# Supplementary material for: Association of ambient air pollutant mixtures with IVF/ICSI-ET clinical pregnancy rates during critical exposure periods
Source: Hum Reprod Open. 2024 Sep 6;2024(3):hoae051. doi: 10.1093/hropen/hoae051 (PMC11412601; doi:10.1093/hropen/hoae051)
Supplement: hoae051_Supplementary_Data [file hoae051_supplementary_data.zip › Supplementary figure.docx]

**Supplementary figure**

| 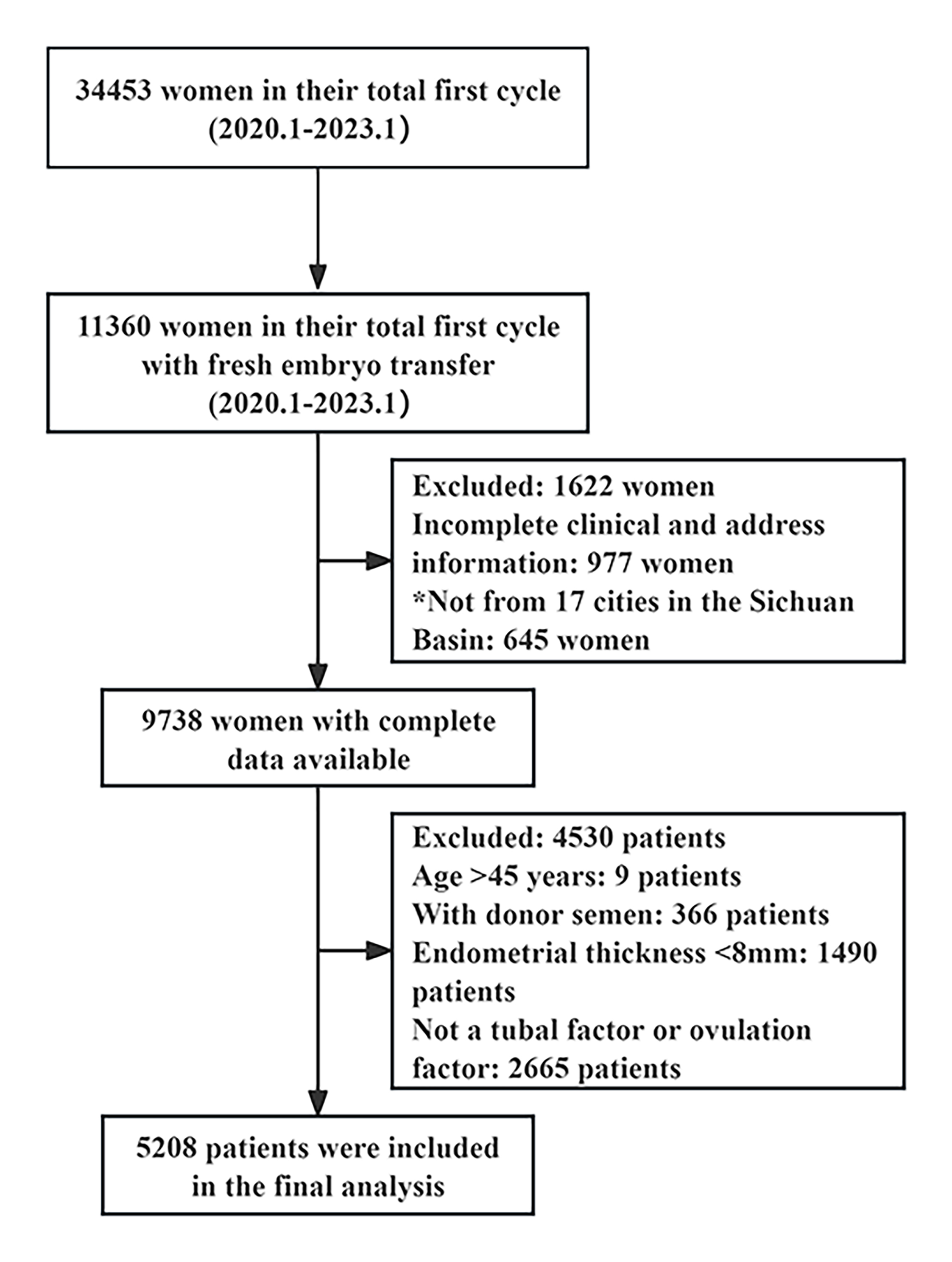 |
| --- |
| **Supplementary Figure S1. Flowchart of patients included in this study.** * Patients were enrolled from 17 cities of the Sichuan Basin including Bazhong City, Chengdu City, Dazhou City, Deyang City, Guang'an City, Guangyuan City, Leshan City, Luzhou City, Meishan City, Mianyang City, Nanchong City, Neijiang City, Suining City, Ya'an City, Yibin City, Ziyang City, and Zigong City. |

|  |
| --- |
| 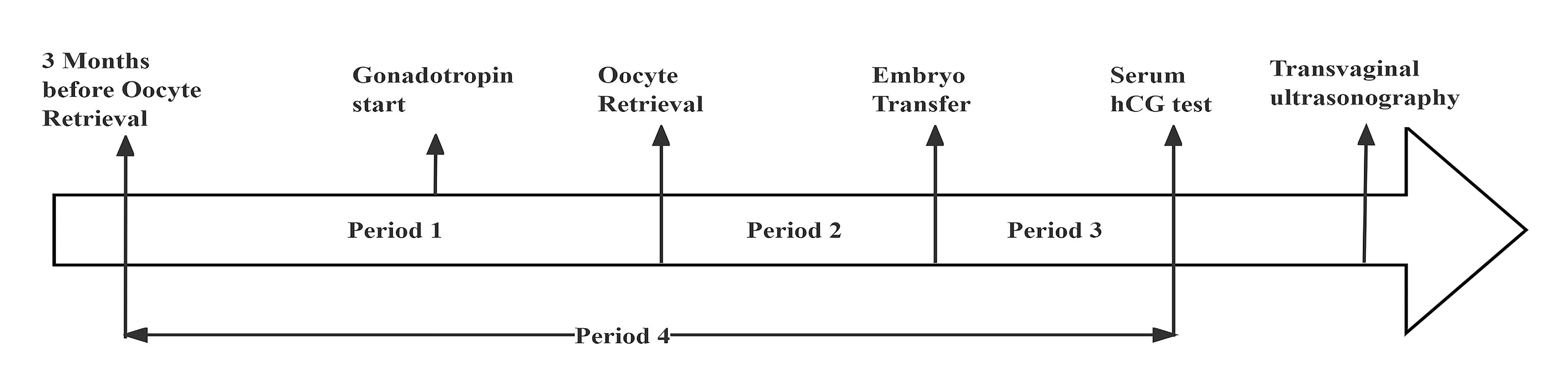 |
| **Supplementary Figure S2. Timeline of in vitro fertilization/intracytoplasmic sperm injection - embryo transfer (IVF/ICSI-ET) periods defined for this study.**  hCG, human chorionic gonadotropin; Period 1, 90 days before oocyte retrieval; Period 2, oocyte retrieval to embryo transfer; Period 3, embryo transfer to serum hCG test; Period 4, 90 days before oocyte retrieval to serum hCG test. |

| 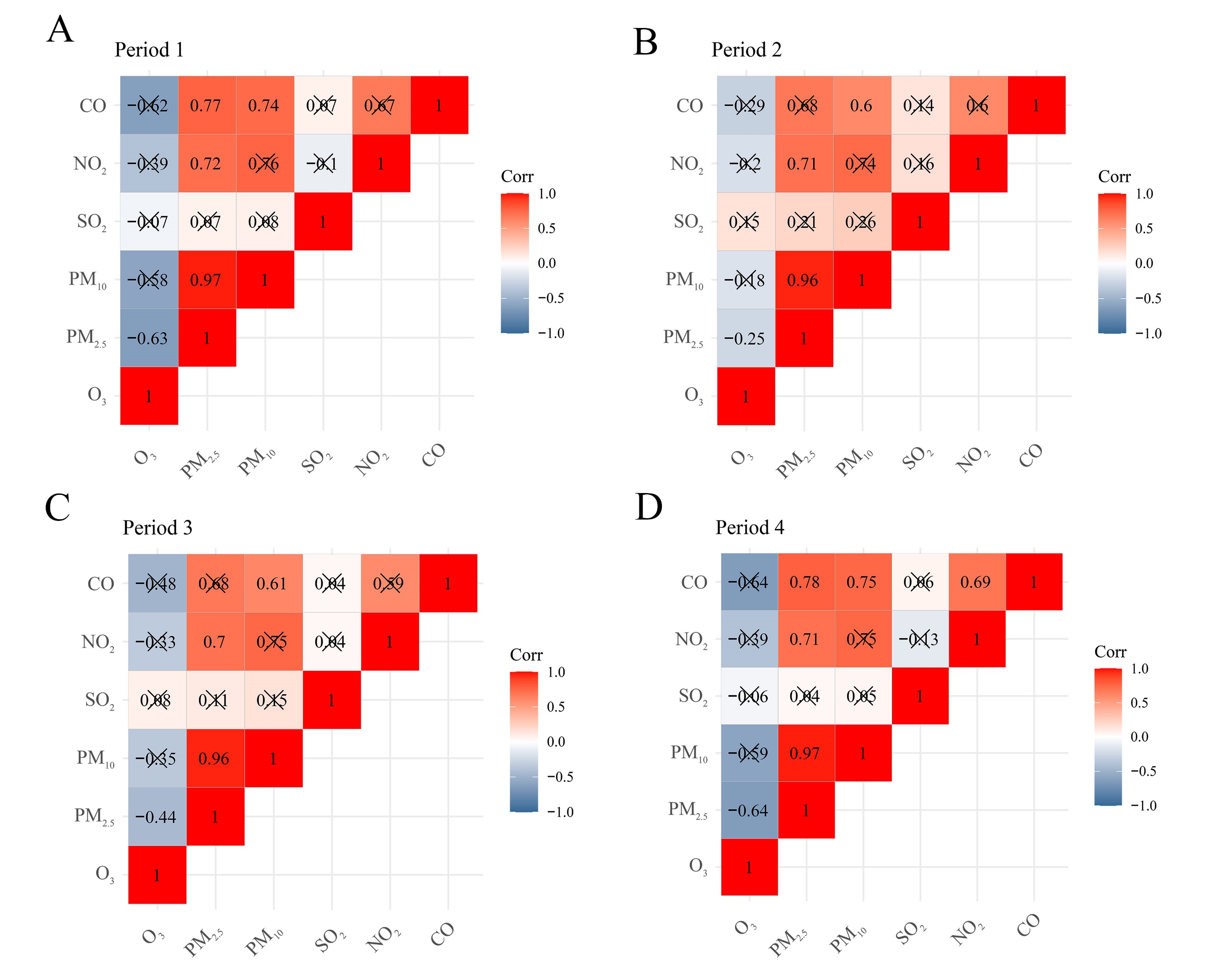 |
| --- |
| **Supplementary Figure S3. Correlation between environmental measurements.**  The Spearman's rank correlation coefficients (*r*_s_) were used to estimate the correlation between ambient air pollutants in each exposure period. ×: indicated no statistical significance (*P*-value ≥ 0.05). PM_2.5_, fine particulate matter (particles ≤ 2.5 µm); PM_10_, inhalable particulate matter (particles ≤ 10 µm); CO, carbon monoxide; NO_2_, nitrogen dioxide; O_3_, ozone, SO_2_, sulfur dioxide; hCG: human chorionic gonadotropin; Period 1, 90 days before oocyte retrieval; Period 2, oocyte retrieval to embryo transfer; Period 3, embryo transfer to serum hCG test; Period 4, 90 days before oocyte retrieval to serum hCG test. |

| 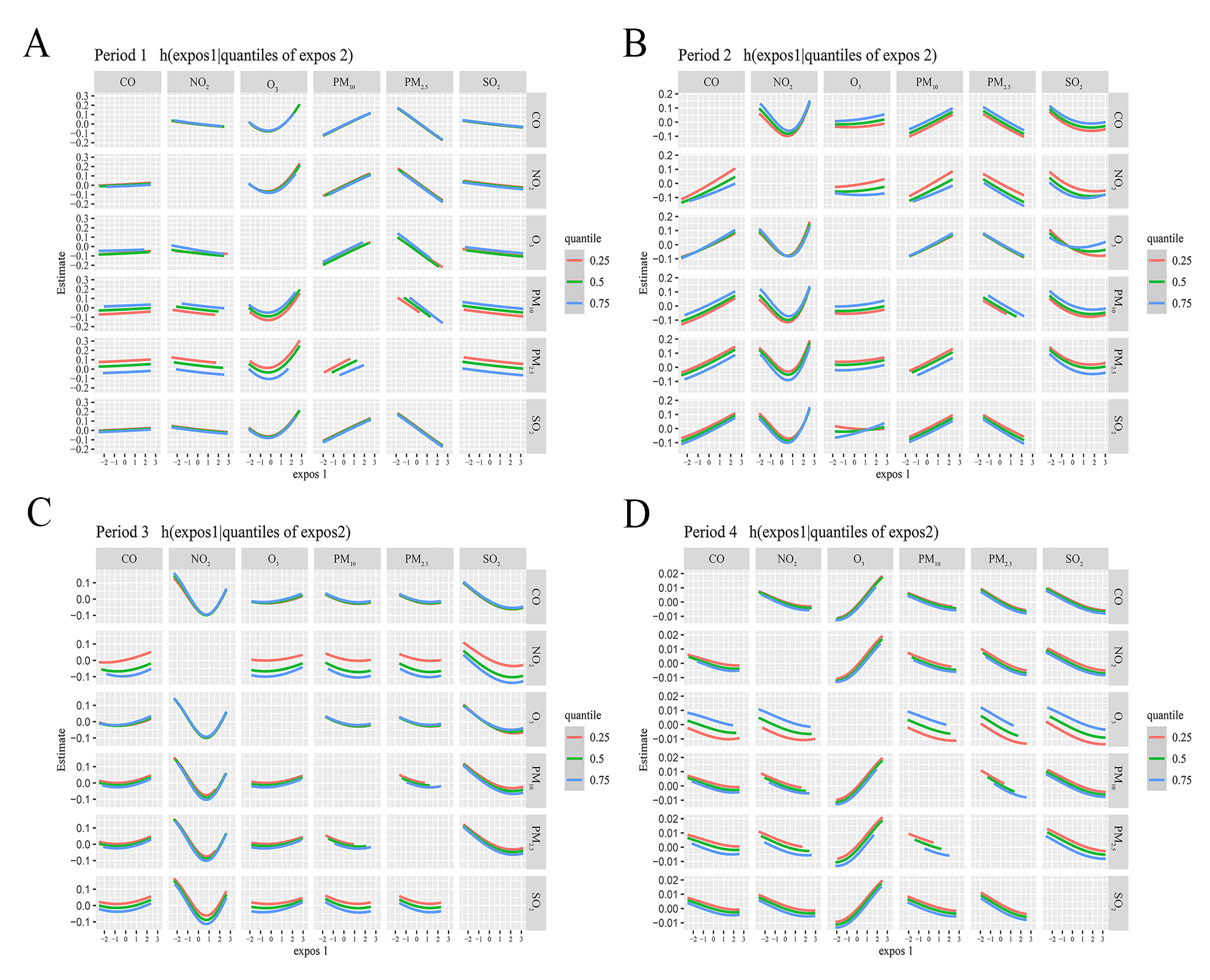 |
| --- |
| **Supplementary Figure S4. The interaction of ambient air pollutants for each exposure period was presented by Bayesian kernel machine regression (BKMR).**  The curves in the figure depicted bivariate dose-response plots for each pollutant when fixed at the percentiles of 25% (red line), 50% (green line), and 75% (blue line), respectively, while keeping other pollutants fixed at the median. PM_2.5_, fine particulate matter (particles ≤ 2.5 µm); PM_10_, inhalable particulate matter (particles ≤ 10 µm); CO, carbon monoxide; NO_2_, nitrogen dioxide; O_3_, ozone, SO_2_, sulfur dioxide; hCG: human chorionic gonadotropin; Period 1, 90 days before oocyte retrieval; Period 2, oocyte retrieval to embryo transfer; Period 3, embryo transfer to serum hCG test; Period 4, 90 days before oocyte retrieval to serum hCG test. |

| 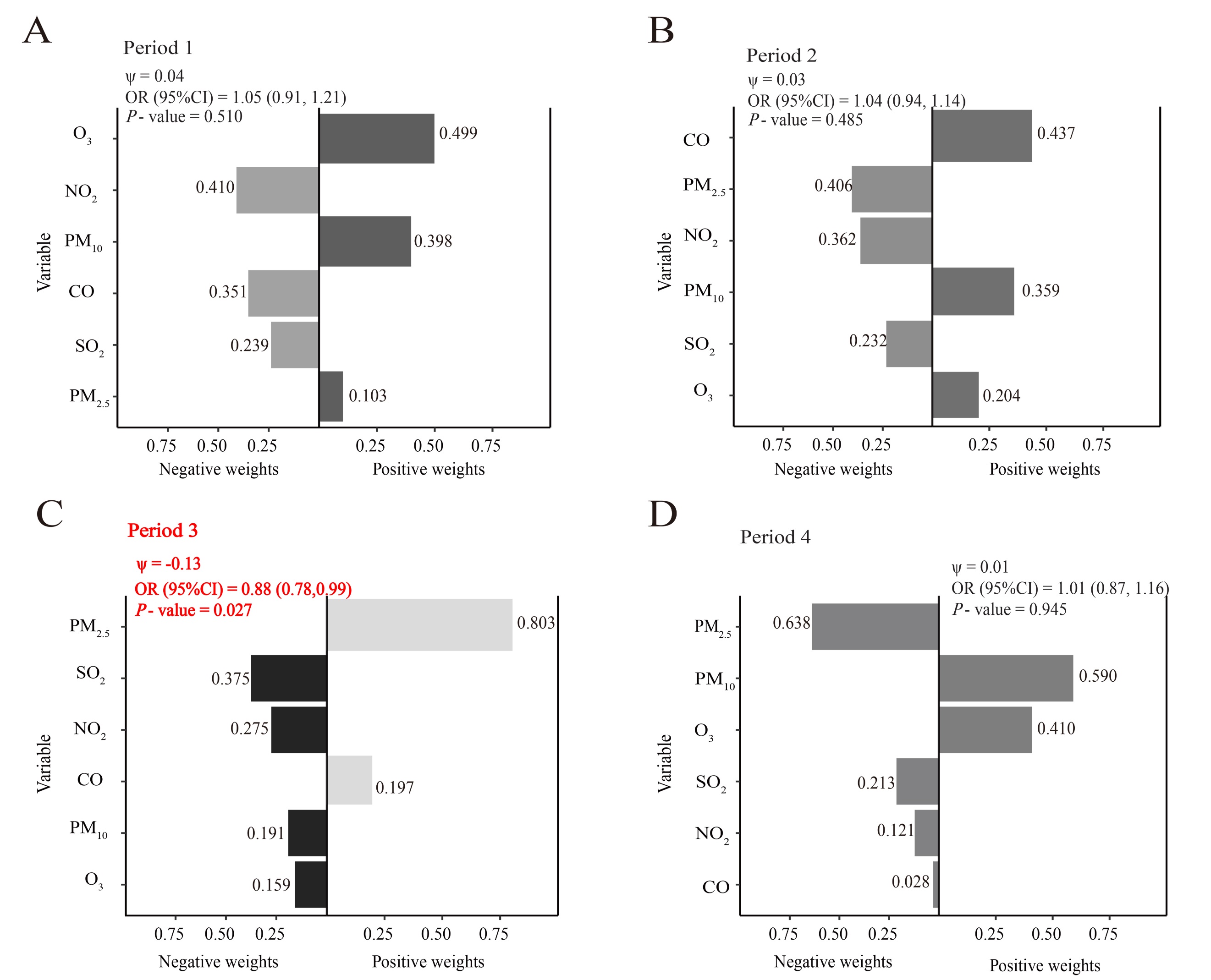 |
| --- |
| **Supplementary Figure S5. Quantile G-Computation (QG-C) regression analysis of mixture air pollution exposure's impact on clinical pregnancy likelihood across various exposure periods in women with tubal infertility.**  Using the QG-C model, after adjusting for age, BMI, stimulation protocol, endometrial thickness on hCG day, total dose of Gn, duration of infertility, infertility type, number of retrieved oocytes, number of embryo transfer, stage of embryo transfer, temperature, and dew point, the relationship between the weights of each pollutant and the likelihood of clinical pregnancy was assessed across four distinct time periods: Period 1 (A), Period 2 (B), Period 3 (C), Period 4 (D). Period 1, 90 days before oocyte retrieval; Period 2, oocyte retrieval to embryo transfer; Period 3, embryo transfer to serum hCG test; Period 4, 90 days before oocyte retrieval to serum hCG test; BMI, body mass index; hCG: human chorionic gonadotropin; Gn, gonadotropin; PM_2.5_, fine particulate matter (particles ≤ 2.5 µm); PM_10_, inhalable particulate matter (particles ≤ 10 µm); CO, carbon monoxide; NO_2_, nitrogen dioxide; O_3_, ozone, SO_2_, sulfur dioxide; ψ, logarithm of the odds ratio; OR, odds ratio, CI, confidence interval. |

| 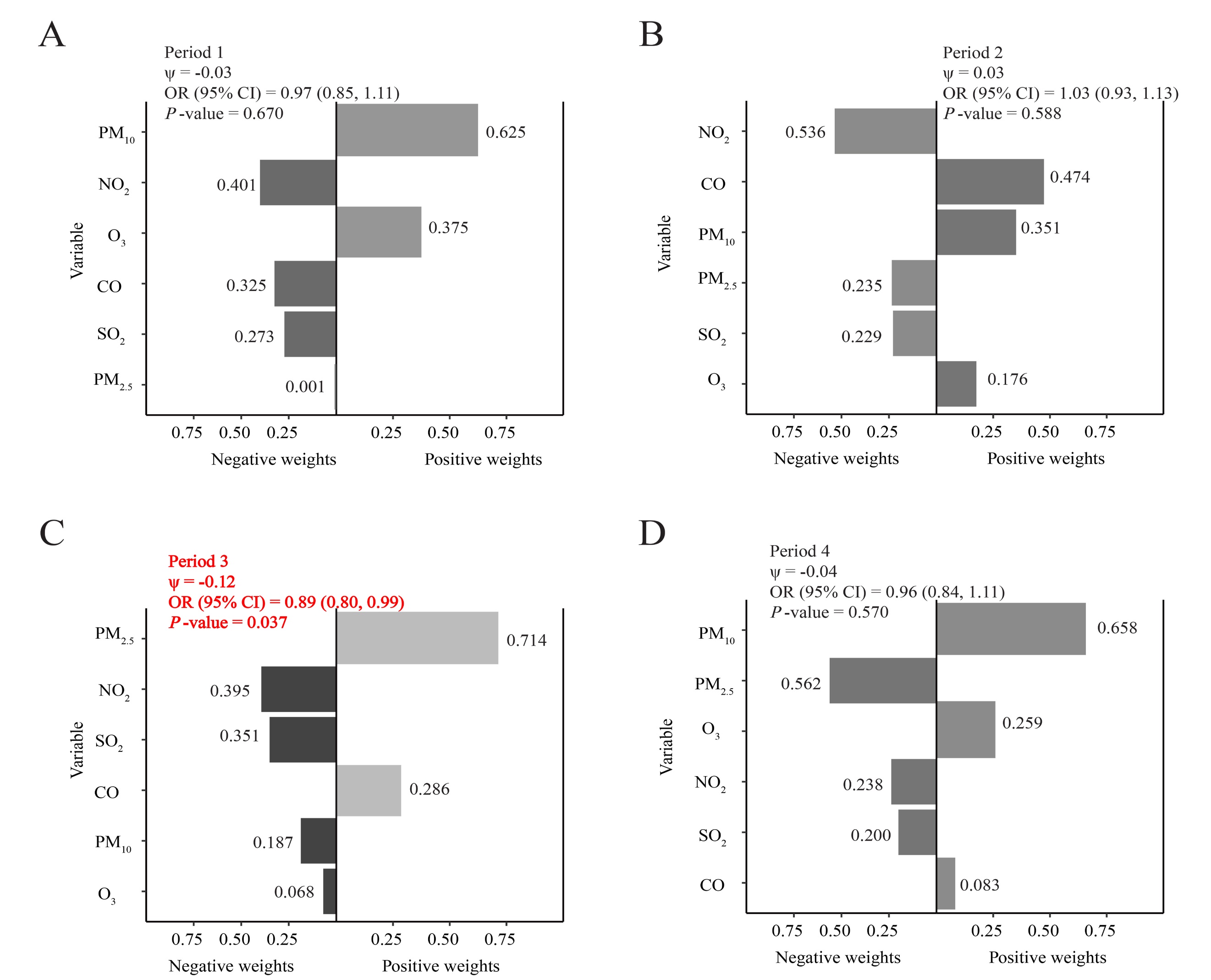 |
| --- |
| **Supplementary Figure S6. Quantile G-Computation (QG-C) regression analysis of mixture air pollution exposure's impact on clinical pregnancy likelihood across various exposure periods with additional adjustment for year of embryo transfer.**  Using the QG-C model, after adjusting for age, BMI, stimulation protocol, endometrial thickness on hCG day, total dose of Gn, duration of infertility, cause of infertility, infertility type, number of retrieved oocytes, number of embryo transfer, stage of embryo transfer, temperature, dew point, and year of embryo transfer, the relationship between the weights of each pollutant and the likelihood of clinical pregnancy was assessed across four distinct time periods: Period 1 (A), Period 2 (B), Period 3 (C), Period 4 (D). Period 1, 90 days before oocyte retrieval; Period 2, oocyte retrieval to embryo transfer; Period 3, embryo transfer to serum hCG test; Period 4, 90 days before oocyte retrieval to serum hCG test; BMI, body mass index; hCG: human chorionic gonadotropin; Gn, gonadotropin; PM_2.5_, fine particulate matter (particles ≤ 2.5 µm); PM_10_, inhalable particulate matter (particles ≤ 10 µm); CO, carbon monoxide; NO_2_, nitrogen dioxide; O_3_, ozone, SO_2_, sulfur dioxide; ψ, logarithm of the odds ratio; OR, odds ratio, CI, confidence interval. |

| 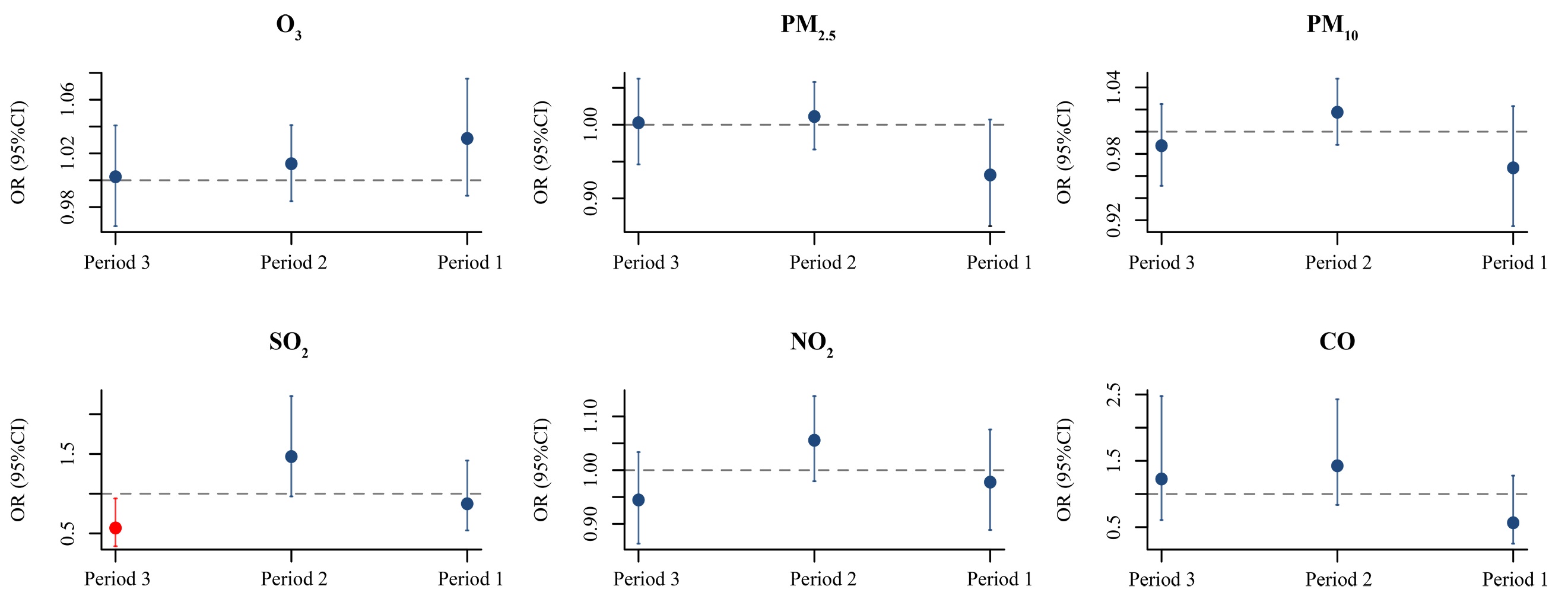 |
| --- |
| **Supplementary Figure S7. Using Distributed Lag Nonlinear Models (DLNM) models to explore the odds ratios of clinical pregnancy likelihood with different periods of pollutant exposure.**  The DLNM model adjusted for age, BMI, stimulation protocol, endometrial thickness on hCG day, total dose of Gn, duration of infertility, cause of infertility, infertility type, number of retrieved oocytes, number of embryo transfer, stage of embryo transfer, temperature, and dew point. In the graph, odds ratios were indicated by circles, each paired with a vertical line representing the 95% confidence interval. Red circles signified statistical significance when the interval did not encompass the value of 1 (*P* - value < 0.05). Conversely, blue circles indicated a lack of statistical significance where the interval included 1 (*P* -value ≥ 0.05). Period 1, 90 days before oocyte retrieval; Period 2, oocyte retrieval to embryo transfer; Period 3, embryo transfer to serum hCG test; Period 4, 90 days before oocyte retrieval to serum hCG test; BMI, body mass index; hCG: human chorionic gonadotropin; Gn, gonadotropin; PM_2.5_, fine particulate matter (particles ≤ 2.5 µm); PM_10_, inhalable particulate matter (particles ≤ 10 µm); CO, carbon monoxide; NO_2_, nitrogen dioxide; O_3_, ozone, SO_2_, sulfur dioxide. |

| 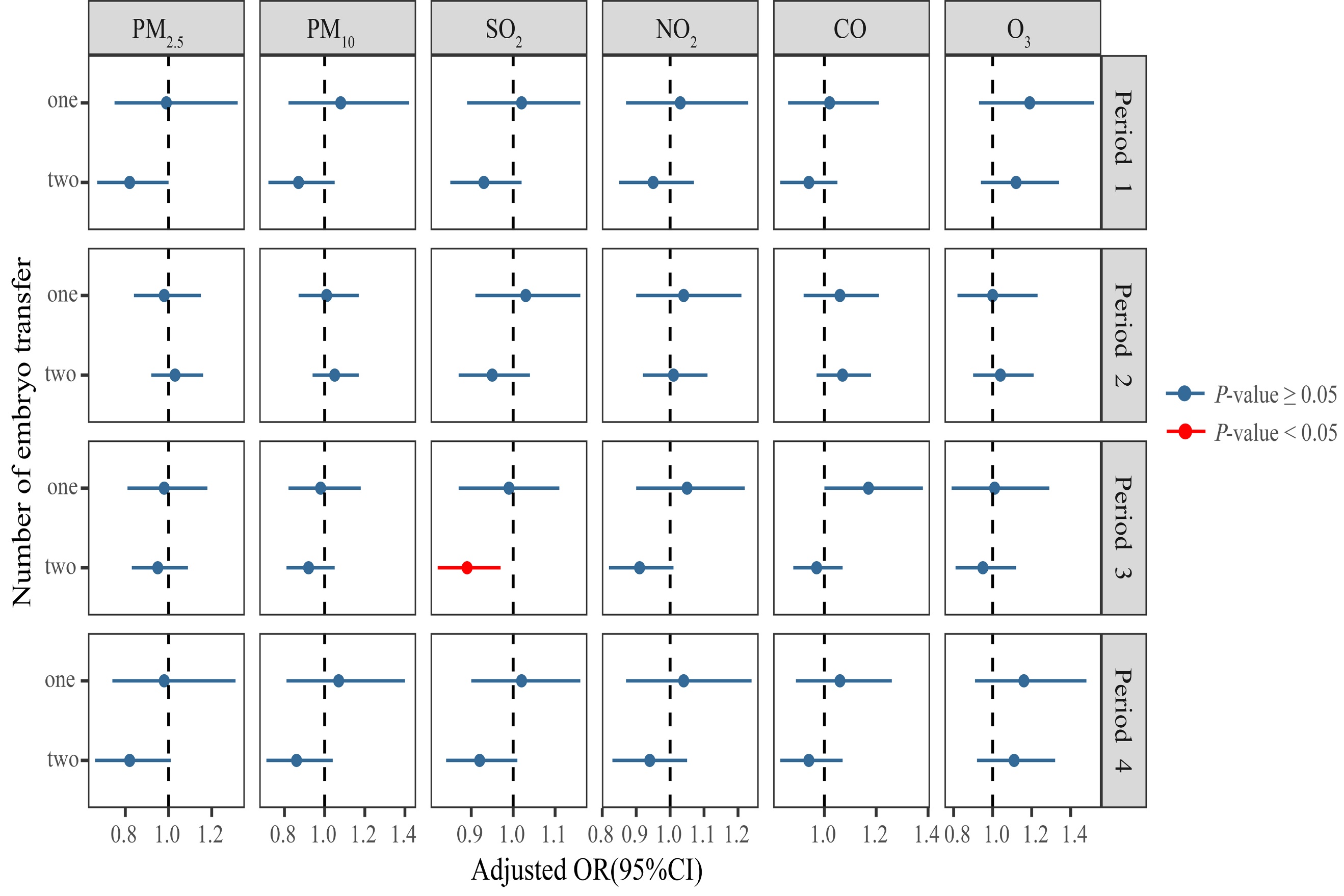 |
| --- |
| **Supplementary Figure S8. Associations between air pollutant exposure and clinical pregnancy at each period stratified by the number of embryo transfer.**  The logistic regression models were adjusted for a range of factors including age, BMI, stimulation protocol, endometrial thickness on hCG day, total dose of Gn, duration of infertility, cause of infertility, infertility type, number of retrieved oocytes, stage of embryo transfer, temperature and dew point. The circles and error bars represented odds ratios (ORs) and 95% confidence intervals (95% CIs), respectively. Significant correlations (*P*- value < 0.05) were shown in red. Period 1, 90 days before oocyte retrieval; Period 2, oocyte retrieval to embryo transfer; Period 3, embryo transfer to serum hCG test; Period 4, 90 days before oocyte retrieval to serum hCG test; BMI, body mass index; hCG: human chorionic gonadotropin; Gn, gonadotropin; PM_2.5_, fine particulate matter (particles ≤ 2.5 µm); PM_10_, inhalable particulate matter (particles ≤ 10 µm); CO, carbon monoxide; NO_2_, nitrogen dioxide; O_3_, ozone, SO_2_, sulfur dioxide. |

| 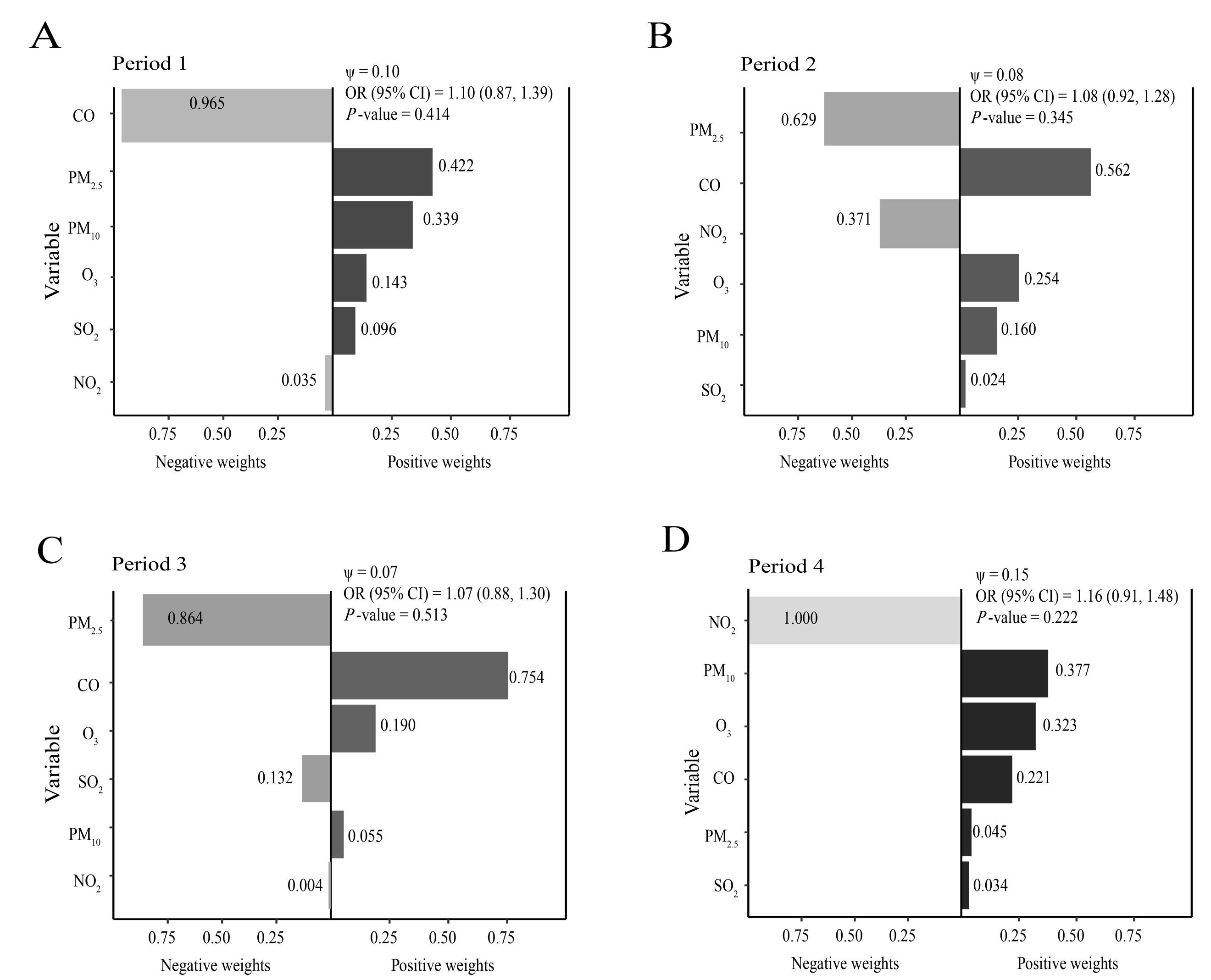 |
| --- |
| **Supplementary Figure S9. Quantile G-Computation (QG-C) regression analysis of mixture air pollution exposure's impact on clinical pregnancy likelihood across various exposure periods in patients receiving one embryo.**  Using the QG-C model, after adjusting for age, BMI, stimulation protocol, endometrial thickness on hCG day, total dose of Gn, duration of infertility, cause of infertility, infertility type, number of retrieved oocytes, stage of embryo transfer, temperature, and dew point, the relationship between the weights of each pollutant and the likelihood of clinical pregnancy was assessed across four distinct time periods: Period 1 (A), Period 2 (B), Period 3 (C), Period 4 (D). Period 1, 90 days before oocyte retrieval; Period 2, oocyte retrieval to embryo transfer; Period 3, embryo transfer to serum hCG test; Period 4, 90 days before oocyte retrieval to serum hCG test; BMI, body mass index; hCG: human chorionic gonadotropin; Gn, gonadotropin; PM_2.5_, fine particulate matter (particles ≤ 2.5 µm); PM_10_, inhalable particulate matter (particles ≤ 10 µm); CO, carbon monoxide; NO_2_, nitrogen dioxide; O_3_, ozone, SO_2_, sulfur dioxide; ψ, logarithm of the odds ratio; OR, odds ratio, CI, confidence interval. |

| 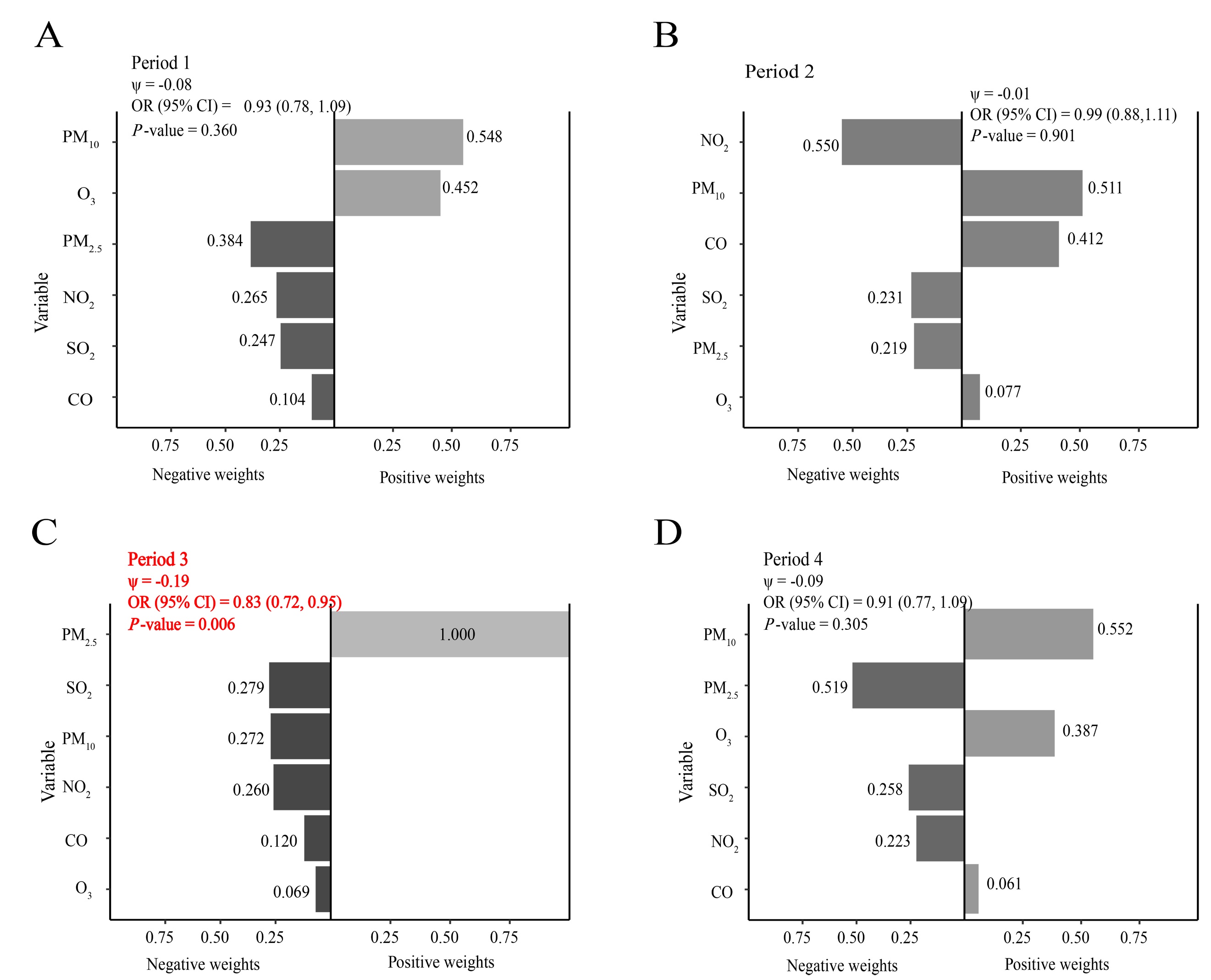 |
| --- |
| **Supplementary Figure S10. Quantile G-Computation (QG-C) regression analysis of mixture air pollution exposure's impact on clinical pregnancy likelihood across various exposure periods in patients receiving two embryos.**  Using the QG-C model, after adjusting for age, BMI, stimulation protocol, endometrial thickness on hCG day, total dose of Gn, duration of infertility, cause of infertility, infertility type, number of retrieved oocytes, stage of embryo transfer, temperature, and dew point, the relationship between the weights of each pollutant and the likelihood of clinical pregnancy was assessed across four distinct time periods: Period 1 (A), Period 2 (B), Period 3 (C), Period 4 (D). Period 1, 90 days before oocyte retrieval; Period 2, oocyte retrieval to embryo transfer; Period 3, embryo transfer to serum hCG test; Period 4, 90 days before oocyte retrieval to serum hCG test; BMI, body mass index; hCG: human chorionic gonadotropin; Gn, gonadotropin; PM_2.5_, fine particulate matter (particles ≤ 2.5 µm); PM_10_, inhalable particulate matter (particles ≤ 10 µm); CO, carbon monoxide; NO_2_, nitrogen dioxide; O_3_, ozone, SO_2_, sulfur dioxide; ψ, logarithm of the odds ratio; OR, odds ratio, CI, confidence interval. |

| 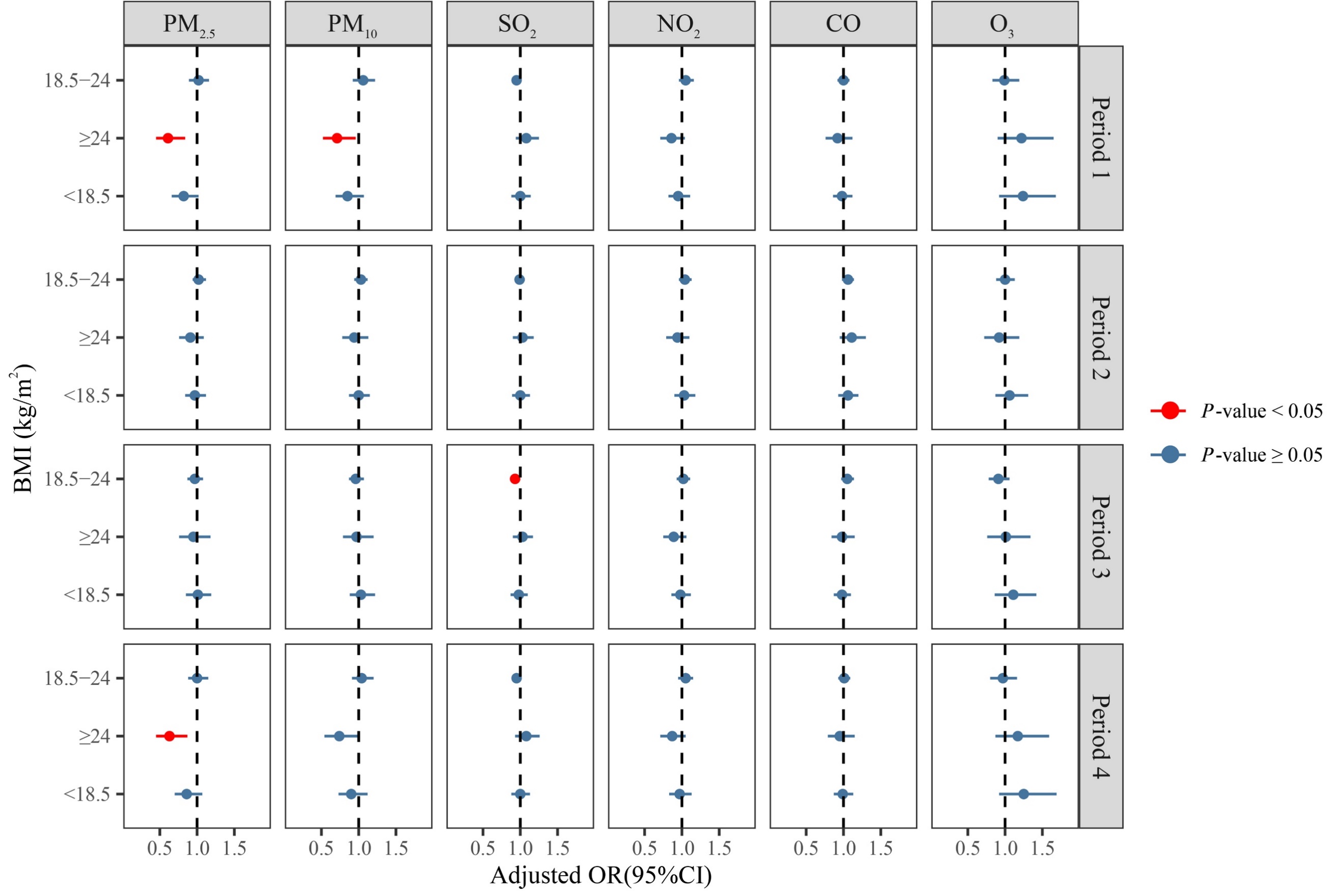 |
| --- |
| **Supplementary Figure S11. Associations between air pollutant exposure and clinical pregnancy at each period stratified by BMI.**  The logistic regression model, adjusted for age, stimulation protocol, endometrial thickness on hCG day, total dose of Gn, duration of infertility, cause of infertility, infertility type, number of retrieved oocytes, number of embryo transfer, stage of embryo transfer, temperature and dew point. The circles and error bars represented odds ratios (ORs) and 95% confidence intervals (95% CIs), respectively. Significant correlations (*P* -value < 0.05) were shown in red. Period 1, 90 days before oocyte retrieval; Period 2, oocyte retrieval to embryo transfer; Period 3, embryo transfer to serum hCG test; Period 4, 90 days before oocyte retrieval to serum hCG test; BMI, body mass index; hCG: human chorionic gonadotropin; Gn, gonadotropin; PM_2.5_, fine particulate matter (particles ≤ 2.5 µm); PM_10_, inhalable particulate matter (particles ≤ 10 µm); CO, carbon monoxide; NO_2_, nitrogen dioxide; O_3_, ozone, SO_2_, sulfur dioxide. |

| 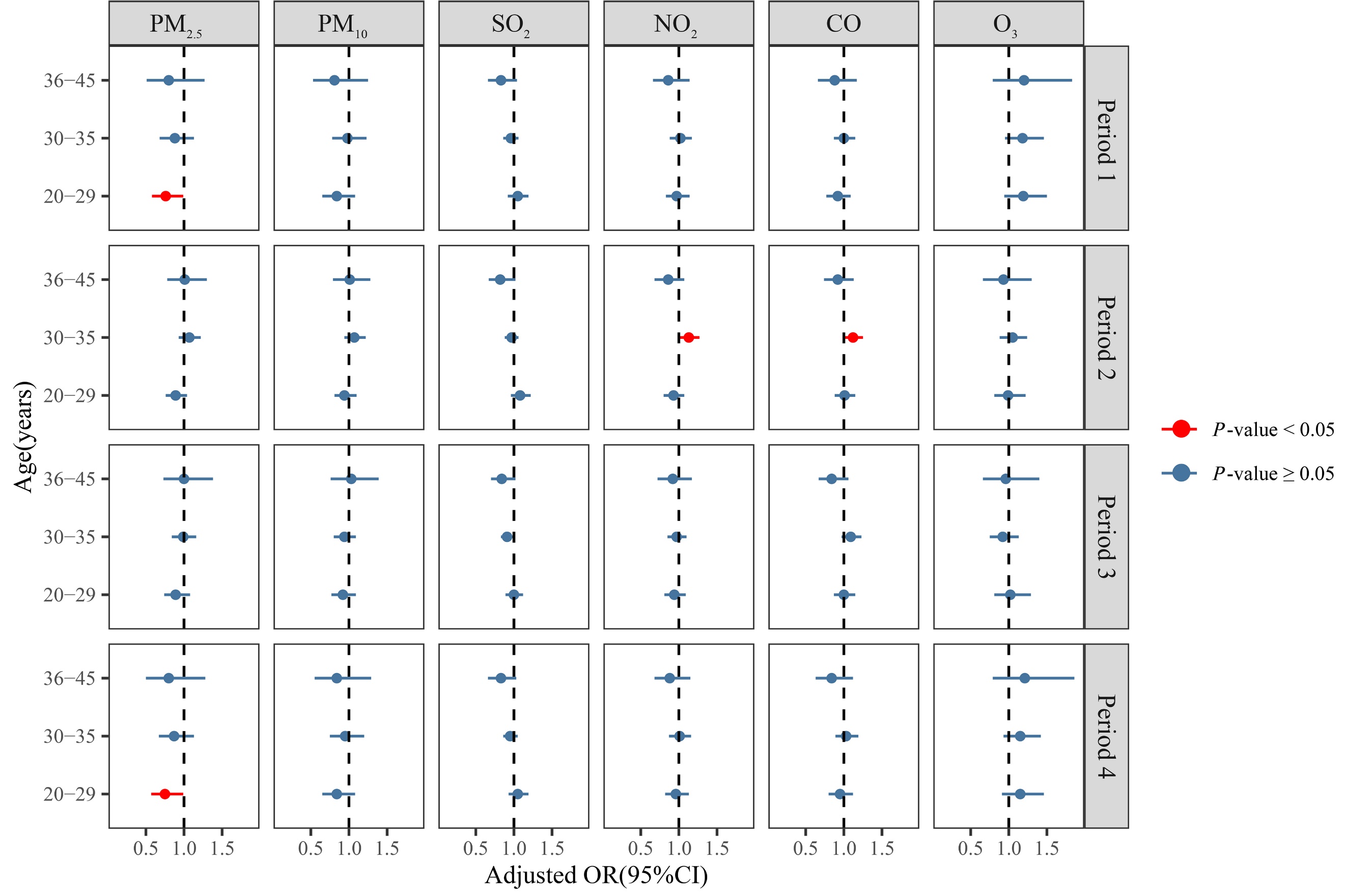 |
| --- |
| **Supplementary Figure S12. Associations between air pollutant exposure and clinical pregnancy at each Period stratified by age.**  The logistic regression model, adjusted for BMI, stimulation protocol, endometrial thickness on hCG day, total dose of Gn, duration of infertility, cause of infertility, infertility type, number of retrieved oocytes, number of embryo transfer, stage of embryo transfer, temperature and dew point. The circles and error bars represented odds ratios (ORs) and 95% confidence intervals (95% CIs), respectively. Significant correlations (*P* -value < 0.05) were shown in red. Period 1, 90 days before oocyte retrieval; Period 2, oocyte retrieval to embryo transfer; Period 3, embryo transfer to serum hCG test; Period 4, 90 days before oocyte retrieval to serum hCG test; BMI, body mass index; hCG: human chorionic gonadotropin; Gn, gonadotropin; PM_2.5_, fine particulate matter (particles ≤ 2.5 µm); PM_10_, inhalable particulate matter (particles ≤ 10 µm); CO, carbon monoxide; NO_2_, nitrogen dioxide; O_3_, ozone, SO_2_, sulfur dioxide. |
