# Supplementary material for: Association of ambient air pollutant mixtures with IVF/ICSI-ET clinical pregnancy rates during critical exposure periods
Source: Hum Reprod Open. 2024 Sep 6;2024(3):hoae051. doi: 10.1093/hropen/hoae051 (PMC11412601; doi:10.1093/hropen/hoae051)
Supplement: hoae051_Supplementary_Data [file hoae051_supplementary_data.zip › Supplementary table.docx]

**Supplementary Table**

| **Supplementary Table S1.** Descriptive of exposure concentrations of ambient air pollution in different periods among the participants in Sichuan basin. | | | |
| --- | --- | --- | --- |
| Pollutants | Period | Mean (SD) [range] | Median (IQR) |
| PM_2.5_ (μg/m^3^) | 1 | 36.55 (15.50) [8.50–74.26] | 33.45 (22.77) |
|  | 2 | 36.19 (20.65) [4.33–82.02] | 30.42 (27.89) |
|  | 3 | 37.12 (18.74) [6.46–79.98] | 32.48 (26.15) |
|  | 4 | 36.65 (14.38) [8.53–73.59] | 34.21 (22.31) |
| PM_10_ (μg/m^3^) | 1 | 57.17 (19.22) [18.35–107.34] | 55.67 (30.45) |
|  | 2 | 57.55 (29.31) [9.13–126.25] | 49.95 (41.11) |
|  | 3 | 57.94 (25.31) [13.76–121.62] | 52.54 (37.04) |
|  | 4 | 57.26 (18.39) [18.58–107.30] | 55.74 (29.38) |
| SO_2_ (μg/m^3^) | 1 | 6.53 (1.67) [2.10–11.95] | 6.34 (2.16) |
|  | 2 | 6.46 (2.27) [1.62–13.30] | 6.16 (2.86) |
|  | 3 | 6.41 (2.01) [1.51–12.78] | 6.21 (2.45) |
|  | 4 | 6.51 (1.64) [2.21–11.89] | 6.31 (2.12) |
| NO_2_ (μg/m^3^) | 1 | 29.31 (8.96) [6.50–55.34] | 28.74 (13.18) |
|  | 2 | 29.44 (12.09) [4.33–60.57] | 27.33 (16.47) |
|  | 3 | 29.28 (10.73) [4.46–57.43] | 27.91 (14.94) |
|  | 4 | 29.30 (8.69) [6.42–55.01] | 28.77 (12.99) |
| CO (mg/m^3^) | 1 | 0.66 (0.11) [0.39–0.93] | 0.65 (0.13) |
|  | 2 | 0.66 (0.16) [0.25–1.05] | 0.64 (0.20) |
|  | 3 | 0.66 (0.13) [0.33–0.98] | 0.64 (0.16) |
|  | 4 | 0.66 (0.10) [0.40–0.93] | 0.65 (0.13) |
| O_3_ 8-h (μg/m^3^) | 1 | 88.06 (29.96) [24.05–172.22] | 88.13 (49.50) |
|  | 2 | 87.02 (41.45) [12.11–201.50] | 81.00 (61.76) |
|  | 3 | 87.04 (37.48) [17.30–197.44] | 86.50 (59.86) |
|  | 4 | 87.77 (28.57) [26.57–159.53] | 87.98 (47.47) |
| Abbreviations: PM_2.5_, fine particulate matter; PM_10_, inhalable PM; CO, carbon monoxide; NO_2_, nitrogen dioxide; O_3_, ozone, and SO_2_, sulfur dioxide; O_3_, ozone; PM, particulate matter; NO_2_, nitrogen; SD, standard deviation; IQR, interquartile range; hCG, human chorionic gonadotropin; Period 1, 90 days before oocyte retrieval; Period 2, oocyte retrieval to embryo transfer; Period 3, embryo transfer to serum hCG test; Period 4, 90 days before oocyte retrieval to serum hCG test. | | | |

| **Supplementary Table S2.** Descriptive of exposure concentrations of meteorological variables in different periods among the participants in Sichuan basin. | | | |
| --- | --- | --- | --- |
| meteorological variables | Period | Mean (SD) [range] | Median (IQR) |
| Temperature (°C) | 1 | 16.41 (6.54) [-4.04, 30.45] | 16.32 (11.85) |
| Temperature (°C) | 2 | 16.20 (7.72) [-6.70, 35.89] | 16.28 (13.67) |
| Temperature (°C) | 3 | 16.12 (7.59) [-6.09, 36.16] | 16.31 (13.37) |
| Temperature (°C) | 4 | 16.36 (6.29) [-4.23, 29.47] | 16.28 (11.61) |
| Dew point (°C) | 1 | 0.46 (13.88) [-11.45, 2.21] | 0.65 (1.35) |
| Dew point (°C) | 2 | 0.50 (24.05) [-35.50, 2.45] | 0.86 (1.32) |
| Dew point (°C) | 3 | 0.43 (18.47) [-19.22, 2.37] | 0.77 (1.34) |
| Dew point (°C) | 4 | 0.46 (13.44) [-10.89, 2.18] | 0.65 (1.30) |
| Abbreviations: SD, standard deviation; IQR, interquartile range; hCG, human chorionic gonadotropin; Period 1, 90 days before oocyte retrieval; Period 2, oocyte retrieval to embryo transfer; Period 3, embryo transfer to serum hCG test; Period 4, 90 days before oocyte retrieval to serum hCG test. | | | |
